# Precise genome-wide mapping of single nucleosomes and linkers in vivo

**DOI:** 10.1186/s13059-018-1398-0

**Published:** 2018-02-09

**Authors:** Răzvan V. Chereji, Srinivas Ramachandran, Terri D. Bryson, Steven Henikoff

**Affiliations:** 1 Division of Developmental Biology, Eunice Kennedy Shriver National Institute for Child Health and Human Development, National Institutes of Health, Bethesda, MD 20892 USA; 2 Howard Hughes Medical Institute and Basic Sciences Division, Fred Hutchinson Cancer Research Center, Seattle, WA 98109 USA

**Keywords:** Chemical cleavage mapping, Linker quantization, Biophysical modeling

## Abstract

We developed a chemical cleavage method that releases single nucleosome dyad-containing fragments, allowing us to precisely map both single nucleosomes and linkers with high accuracy genome-wide in yeast. Our single nucleosome positioning data reveal that nucleosomes occupy preferred positions that differ by integral multiples of the DNA helical repeat. By comparing nucleosome dyad positioning maps to existing genomic and transcriptomic data, we evaluated the contributions of sequence, transcription, and histones H1 and H2A.Z in defining the chromatin landscape. We present a biophysical model that neglects DNA sequence and shows that steric occlusion suffices to explain the salient features of nucleosome positioning.

**Electronic supplementary material:**

The online version of this article (10.1186/s13059-018-1398-0) contains supplementary material, which is available to authorized users.

## Background

Nucleosomes are the basic units of DNA packaging in eukaryotes. They contain ~ 147 bp of DNA, wrapped around a histone octamer, in about 1.7 superhelical turns [1–3]. Nucleosomes have multiple roles inside the nucleus. In addition to DNA packaging, nucleosome organization plays a crucial role in controlling DNA accessibility of many DNA-binding proteins to regulatory elements on the chromosomes. Nucleosome positions influence gene expression regulation [4–7], DNA replication [8, 9], DNA repair [8, 10], and DNA recombination [11]. The importance of nucleosome position in understanding these processes has fueled a debate as to whether the position of a nucleosome is determined in part by inherent sequence preferences [12–16]. The most generally accepted view is that nucleosome positioning is determined by a combination of DNA sequence, ATP-dependent remodeling enzymes, transcription factors, and elongating RNA polymerases [15]. However, the validity of this paradigm depends on the accuracy and precision with which the in vivo positions of nucleosomes are mapped.

To map nucleosome positions, the chromatin fiber is typically digested with micrococcal nuclease (MNase) followed by high-throughput sequencing (MNase-seq). MNase generates a wide range of fragment sizes even at well-positioned nucleosomes due to unwrapping dynamics of DNA at the entry and exit of the nucleosome compared to the DNA near the dyad axis. Fragments that range in size between 130 and 160 bp may represent intact nucleosomes, where the variation in the length of DNA that wraps around a histone octamer could represent internal cleavages on one end and linker cleavage on the other end. Thus, due to the very nature of MNase cleavage, there is a degree of “fuzziness” in the nucleosome positions determined by MNase-seq.

Linker DNA is about 25 times more susceptible to MNase cleavage, compared to nucleosomal DNA [17], implying that after an extensive digestion, most of the linker DNA will be digested and the remaining DNA will be highly enriched in mononucleosomal fragments. However, MNase digestion produces DNA fragments that are protected both by nucleosomes and by non-histone DNA-binding proteins [18]. MNase also has a strong DNA sequence specificity [19–21]. Furthermore, measuring nucleosome occupancy using MNase has been a problem, and questions have been raised about the degree to which MNase protection measures nucleosome occupancy as opposed to DNA accessibility [22].

Alternative methods have been introduced that promise to circumvent these issues involving the use of MNase to map nucleosomes [23, 24]. In the H4S47C cleavage mapping method [23], substitution of a cysteine for a serine at position 47 of histone H4 converts it into a site-specific nuclease by coupling the cysteine to phenanthroline ex vivo. The phenanthroline chelates a copper ion, which in the presence of peroxide cleaves DNA in its local vicinity. Cleavages due to H4S47C-phenanthroline-Cu^+^ occur close to the pseudo-dyad axis, where the DNA has the least mobility. The ends of the resulting DNA fragments approximate very well the positions of the dyads corresponding to the nucleosomes that produced the cleavage. Thus, the H4S47C cleavage method maps nucleosome dyad positions with base-pair resolution. Initially, this method was used to map nucleosomes in vitro [3, 25]. More recently, this method was successfully applied in vivo in *Saccharomyces cerevisiae* [23, 26], *S. pombe* [27, 28], and mouse embryonic stem cells [29].

H4S47C-anchored chemical cleavage occurs at positions ±1 and ±6 bp from the nucleosomal center. This results in a fragment that spans from one side of the dyad axis of a nucleosome to the dyad axis of the next nucleosome or to a non-specific cleavage at a nucleosome-depleted region (NDR) due to free phenanthroline. The pattern of cleavages around the dyad axis was used to develop a Bayesian deconvolution algorithm in order to infer the most likely positions for the nucleosome centers [23]. Thus, in H4S47C cleavage mapping, the nucleosome positions were derived by considering clusters of many cleavages originating from different cells and predicting the most probable nucleosome locations. However, background cleavages in nucleosome-depleted and linker regions can complicate the use of the method for determining nucleosome occupancy. Moreover, nucleosomes can form at different alternative positions in different cells [30–32], which motivates the development of a method that maps individual nucleosomes rather than relying on inferred coordinates for the most probable location of their centers.

Here we introduce an alternative chemical cleavage method that avoids the complications inherent in H4S47C cleavage mapping. With H4S47C cleavages, the fragment between two cleavages in a single nucleosome is at most 12 bp and is too short to be part of the sequencing library. We hypothesized that moving the cysteine to a DNA-proximal position on a histone that is farther away from the dyad would release a fragment long enough to be sequenced and uniquely mapped. This fragment would represent a single nucleosome and directly indicate that single nucleosome’s position at base-pair resolution. In this study, we describe a new histone mutant, H3Q85C, which when present in two copies in the nucleosome releases a 51-bp DNA fragment from each nucleosome following a chemical cleavage reaction. This 51-bp fragment from a single yeast cell enables the direct and precise assignment of the single nucleosome position. Using H3Q85C cleavage data, we have mapped nucleosome dyad positions, nucleosome occupancies, NDRs, and linker lengths with unprecedented accuracy. Using these data, we resolve features of the chromatin landscape with high precision and propose a biophysical model of nucleosome organization that predicts the major features of nucleosome positioning despite ignoring DNA sequence.

## Results

### H3Q85C cleavage mapping precisely locates dyads of single nucleosomes

**Fig. 1:**
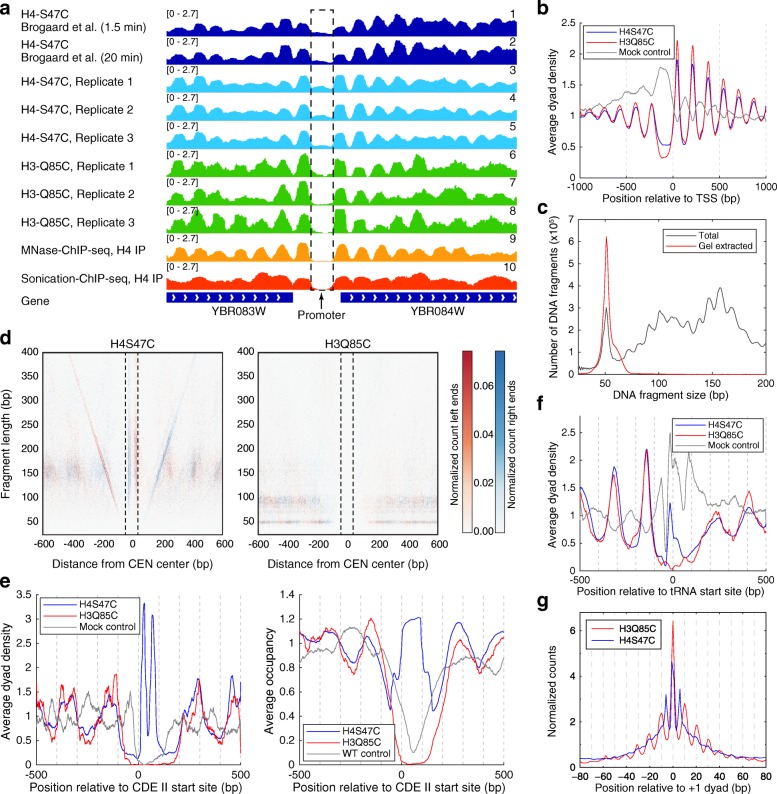
Concurrent chemical cleavages of H3 histones eliminate the background noise in the chemical mapping experiments. **a** Integrative Genomics Viewer browser snapshot illustrating nucleosome maps obtained by different techniques. Previous chemical maps, using mutated histones H4S47C (tracks 1–5), are affected by increased background of non-specific cleavages produced by free phenanthroline. Tracks 1 and 2 show the nucleosome occupancy inferred from H4S47C cleavages reported by [23] by symmetrically extending each cleavage position to a footprint of 101 bp (to emphasize the linker positions) between neighboring nucleosomes. Tracks 3–5 show similar data from [26]. The background from the promoter of YBR084W is strongly reduced when we consider only concurrent chemical cleavages, both produced by H3Q85C (tracks 6–8). Tracks 6–8 show the occupancy produced by symmetrically extending the ~ 50-bp fragment centers to a footprint of 101 bp. The absence of histones from the promoter is confirmed by two alternative chromatin immunoprecipitation sequencing (ChIP-seq) methods (tracks 9 and 10) (data from [51, 60]). To make the occupancy profiles comparable, the MNase-ChIP-seq nucleosome map was generated by symmetrically extending the position of the dyad to a footprint of 101 bp. For sonication-ChIP-seq profiles, the occupancy was computed by stacking all the fragments with length between 50 and 200 bp, as in this case the centers of the fragments do not necessarily represent nucleosome dyads. **b** Average dyad distributions obtained from our H3Q85C cleavage experiment show reduced background noise compared to the map obtained from the H4S47C cleavage experiment. **c** Length distribution of DNA fragments before and after gel filtration. **d** Left-right V-plot display of chemical cleavages, where the *X*-axis shows the position of the center of all 16 aligned centromeres and the *Y*-axis shows the length of each fragment; *red pixels* represent the left fragment ends and *blue pixels* represent the right ends. The two edges of the ~ 80-bp centromere DNA element II (CDEII) are indicated by *dotted lines*. For H4S47C, the *red* and *blue vertical features* between the dotted lines imply that cleavages occur at two distinct positions within CDEII over the population. However, for H3Q85C, no cleavages are seen within the centromeric region, indicating that centromeric nucleosomes do not contain H3 histones. **e** Cleavage density (*left*) and average occupancy (*right*) plots of the data shown in **d**, comparing H4S47C and H3Q85C cleavage data. Mock control data were obtained by phenanthroline treatment and cleavage reactions performed using wild-type cells as described [26]. **f** Cleavage density plots comparing H4S47C, H3Q85C, and Mock control cleavage data over aligned transfer RNA (tRNA) genes. **g** The preferred rotational positions are more evident in the H3S47C cleavage data, even when comparing the nucleosome positions called from the H4S47C cleavage experiment. All dyad distributions were normalized such that the average dyad density equals 1 for every chromosome

H4S47C positions are derived by averaging many cleavages. There are also non-specific cleavages in linkers and NDRs due to free phenanthroline, contributing to noise in H4S47C-anchored cleavage mapping (Fig. 1a, tracks 1–5). To obtain direct measurements of dyad positions and reduce the noise due to non-specific cleavages, we tested mutants at positions farther away from the dyad so that two cleavages on either side of the dyad would give rise to a fragment that is long enough to be sequenced. We found that H3Q85C cleaves nucleosomal DNA and releases 51-bp fragments, as expected from the position of this residue close to the DNA minor groove based on the crystal structure of the nucleosome (Additional file 1: Figure S1). Cleavages on either side of the dyad of a nucleosome result in a 51-bp fragment and drastically reduce the noise due to non-specific cleavages. As each 51-bp fragment arises from a single nucleosome, its midpoint corresponds to the precise position of a nucleosome and directly informs about the distribution of nucleosome centers on the genome without the need to average cleavages from multiple nucleosomes. By using 51-bp fragments generated by H3Q85C cleavages for mapping rather than the linker-spanning fragments generated by H4S47C cleavages, we avoid the background due to cleavages within linkers, which is especially important for distinguishing regions that are merely nucleosome-depleted from those that are virtually nucleosome-free (Fig. 1a; compare tracks 6–8 with tracks 1–5). This reduction in noise is reflected in the average nucleosome distribution relative to the transcription start site (TSS) (Fig. 1b) by the reduced nucleosome density at promoters and increased peak heights at the typical nucleosome positions. Wild-type control cells treated similarly to the mutants show a high density of cleavages at TSSs and linker regions, evidence that the elevated level of cleavages over TSSs using the H4S47C mutant is caused by free phenanthroline.

Although, in addition to intranucleosomal fragments (~51 bp long), H3Q85C cleavages also produce longer internucleosomal fragments, we can easily purify the intranucleosomal fragments using polyacrylamide gel electrophoresis (PAGE) (Fig. 1c). With this increased sensitivity in detecting H3-containing nucleosomes, we asked if this data set could clarify nucleosome occupancy at other genomic landmarks. One model for the budding yeast centromere posited a Cse4-H3 heterotypic nucleosome [33]. With the new H3 localization data, we are able to directly interrogate the presence of histone H3 at yeast centromeres and directly distinguish between these alternative models. When we compared the heat map of fragment ends mapping around the ~ 80-bp centromere DNA element (CDEII) sequence of all yeast chromosomes from our previous H4S47C data [26] and our present H3Q85C data, we observed striking differences (Fig. 1d). The “railroad track” in the H4 data denotes the precise positioning of H4S47C at the centromere. In contrast, we observe no cleavages at the centromere in the H3 data, but uniform coverage of reads adjacent to the centromere, as seen also by averaging the fragments relative to CDEII (Fig. 1e). Thus, H3Q85C mapping rules out the presence of H3 at yeast centromeres. Similarly, at the transfer RNA (tRNA) genes, we see a striking nucleosome depletion in H3Q85C maps (Fig. 1f), whereas H4S47C maps show a high level of cleavages that were likely generated by free phenanthroline (Fig. 1f). Finally, the higher signal-to-noise ratio of H3Q85C enables us to discern alternate rotational positions for nucleosomes defined by H4S47C mapping. We plotted the distribution of nucleosome dyads deduced either from the centers of ~ 51-bp fragments produced by H3Q85C cleavages, or from the individual cleavages produced by H4S47C, relative to the +1 nucleosome positions defined using published H4S47C nucleosome calls (Fig. 1g). Not only did we observe a higher signal at the called dyad itself, but we also observed at least three alternate rotational positions upstream and downstream of the +1 dyad that are invisible to H4S47C mapping. In summary, relying on two intranucleosomal cleavages in H3Q85C mapping enables the generation of a superior nucleosome map in budding yeast.

### H3Q85C cleavage mapping identifies rotational positioning preferences of all nucleosomes in the genome

**Fig. 2:**
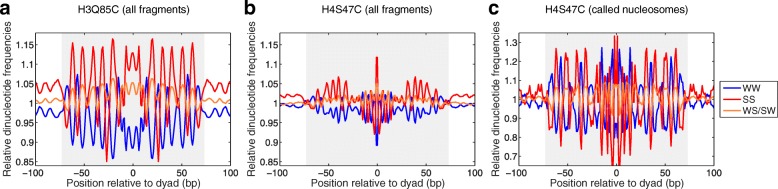
H3Q85C cleavages map rotational positions genome-wide. Nucleosome dyad positions based on raw H3Q85C (**a**) and H4S47C (**b**) cleavage data were aligned and dinucleotide frequencies were mapped at each base pair position. The genome-wide H4S47C rotational positioning signature is irregular and much weaker than the H3Q85C signal. A strong and more regular positioning signature (**c**) is obtained for H4S47C data by using the “called” subset H4S47C nucleosome dyads based on stringent cutoffs [23]

Within the DNA sequences that are wrapped around the histone octamer, there is a compositional preference for WW dinucleotides (AA, TT, AT, or TA) to occur where the minor grooves of DNA directly interact with histones, while SS dinucleotides (CC, GG, CG, or GC) occur where the major grooves of DNA are facing the histones [15]. These alternating DNA sequences have a higher affinity for nucleosome formation [34], and they have been observed among the nucleosomal sequences in different organisms [35–37]. Evolution of this weak compositional preference has a structural rationale, in which more bendable sequences tend to be in contact with the histones every 10 bp and less bendable sequences tend to be solvent-exposed, to facilitate the tight wrapping around the nucleosome core [15]. Originally described using only 177 MNase-generated chicken nucleosomes [36], this enrichment has been confirmed for the yeast genome using MNase-seq [38] and is also seen using H4S47C-anchored cleavage [23]. These analyses are based on called nucleosome positions, which represent a small subset of nucleosomes that can be identified based on their high signal-to-noise ratio using these methods. However, with H3Q85C cleavage mapping, each ~ 51-bp fragment identifies one unique nucleosome without requiring any additional filtering or statistical inference. Thus, we calculated the dinucleotide preference based on every ~ 51- bp fragment. We observed that nucleosome centers determined by H3Q85C-anchored cleavage mapping also show a cumulative rotational phasing of WW and SS dinucleotides (Fig. 2a). The power of this analysis is apparent when we compare the WW/SS distributions generated from every read of H3Q85C mapping to WW/SS distributions generated from every read of H4S47C mapping (Fig. 2b). The WW/SS distributions appear stronger and phased for H4S47C mapping only when limited to the strong “called” nucleosome positions (Fig. 2c), representing a subset of the nucleosomes compared to H3Q85C mapping, which in principle represents all nucleosomes genome-wide.

### Precise identification of NDRs and flanking nucleosomes

Most yeast genes have a stereotypical nucleosome organization in their promoters, characterized by an NDR just upstream of the TSS, which is flanked by regular arrays of nucleosomes [39–43]. Various factors are partly responsible for this organization: (1) non-histone proteins bind to promoters and compete with nucleosome formation [44–46]; (2) some DNA sequence motifs present in promoters are unfavorable for nucleosome formation [47, 48]; (3) chromatin organization is disrupted by transcription [49–51] and replication [52–55]; and (4) nucleosome positioning is maintained by chromatin remodelers [56–60].

**Fig. 3:**
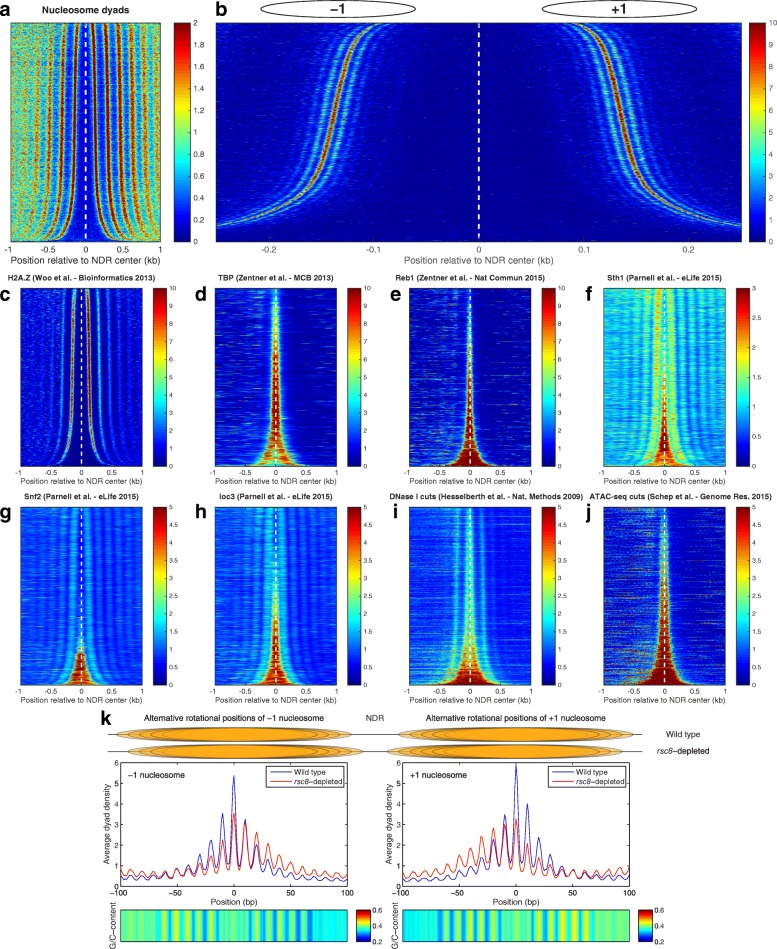
Characterization of yeast promoters. **a** Heat map of the nucleosome dyad distribution at yeast promoters. Each row represents a 2-kb window focused on the NDR centers of yeast genes, which are sorted according to the NDR width. **b** Zoomed-in view of a 500-bp region around the NDR center, including only the +1 and –1 nucleosomes of all yeast genes. The high resolution of the nucleosome positioning data allows the identification in the ensemble of cells of alternative rotational positions (*parallel sigmoidal stripes* corresponding to +1 and –1 nucleosomes), which differ by multiples of the helical twist. **c** NDRs are flanked by H2A.Z-containing nucleosomes (data from [61]). NDRs are generally bound by TATA-binding protein (**d**) (TBP, data from [62]), Reb1 (**e**) (data from [63]), remodelers RSC, Snf2, and ISW1 (**f-h**) (data from [64]). NDRs are accessible to DNase I (**i**) (data from [65]) and transposase Tn5 (**j**) (data from [66]). **k** In rsc8-depleted cells, nucleosomes +1 and –1 shift by a multiple of the helical twist, maintaining the same rotational setting. Heat maps showing the average G/C content of the preferred dyad locations. MNase-seq data from [58]

The lack of background cleavages at promoters using H3Q85C mapping (Fig. 1a, tracks 6–8) facilitates identification of the exact locations of every NDR and the corresponding flanking nucleosomes (+1 and –1 nucleosomes). Using a previously described procedure [60], we identified all NDRs and the precise locations of +1 and –1 nucleosomes in *S. cerevisiae* (Additional file 2: Table S1). We then aligned all NDRs and sorted them according to their widths (Fig. 3a, b). Figure 3b demonstrates the high resolution of our nucleosome positioning data, in which we can distinguish even alternative rotational positions that are occupied by nucleosomes in different cells. These alternative positions from different cells differ by the magnitude of the DNA helical twist (~10 bp), so they can only be distinguished if the accuracy of the nucleosome positioning data has a resolution < 5 bp (the separation between a maximum and a minimum in the dyad distribution), which is not attainable using MNase-seq.

Analyzing other published data sets by aligning the promoters in the same way, we observed that most of the NDRs are flanked by H2A.Z-containing nucleosomes (Fig. 3c) (data from [61]) and are bound by a multitude of proteins, e.g., TATA-binding protein (TBP, data from [62]), Reb1 (data from [63]), and Sth1, Snf2, Ioc3 (data from [64]) (Fig. 3d-h). As expected, the NDRs are also DNase I hypersensitive (data from [65]) and accessible to transposase Tn5 (data from [66]) (Fig. 3i, j), indicating that our identification of NDRs captures known properties of yeast promoters.

The precise positions of +1 and –1 nucleosomes can be used to get insights about important processes that involve nucleosomes, e.g., to study the nucleosome shifts, nucleosome eviction, and spacing changes produced by chromatin remodelers with high resolution at the genome-wide scale, or to study the precise locations of pausing when polymerases transcribe through nucleosomes. As a proof of concept, we re-analyzed the nucleosome shifts produced through depletion of RSC from yeast cells. Previously, it was shown that in *rsc8*-depleted cells, nucleosomes near the gene promoters invade the NDR, reducing its width [58], although the precise mechanism of nucleosome displacement remains unknown. We wondered whether the +1 and –1 nucleosomes shift toward the NDRs by a random number of base pairs, or whether they change their locations among the alternative rotational positions observed in Fig. 3b by shifting by an integral number of helical twists along the DNA, thus maintaining the same rotational setting. Using the reference positions that we identified for +1 and –1 nucleosomes in wild-type cells and the MNase-seq data for *rsc8*-depleted and wild-type cells [58], we made an unexpected discovery. The +1 and –1 nucleosomes preferentially occupy the same rotational positions, both in wild-type and in *rsc8*-depleted cells (Fig. 3k), and it is only the relative occupancy of each of these alternative positions that changes in the mutant cells. This suggests that RSC maintains the proper NDR width by shifting nucleosomes in steps equal to the helical twist, maintaining at the same time the preferred rotational orientation of the DNA relative to the histone core. Aligning all DNA sequences of +1 and –1 nucleosomes, we observed that the average G/C content of these two loci is also oscillatory (Fig. 3k, bottom heat maps). This suggests that at the single-nucleosome level, the histone core is able to sample an ensemble of favorable rotational positions, determined by the underlying DNA sequence, and chromatin remodelers affect the stochastic process of selecting the individual positions of nucleosomes in each cell.

**Fig. 4:**
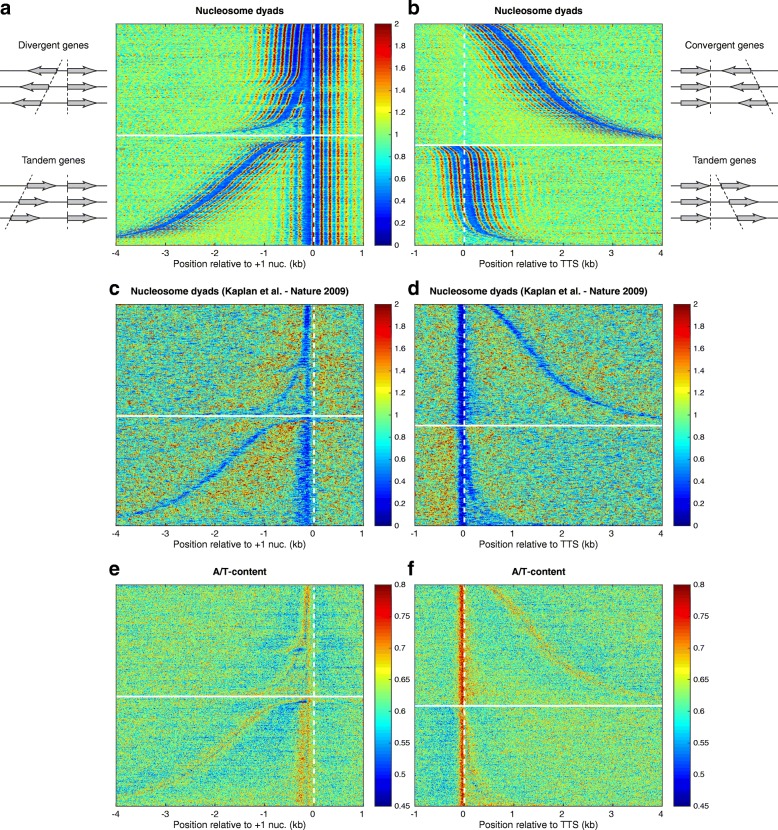
Nucleosome phasing patterns are characteristic of 5’ but not 3’ gene ends. **a** Nucleosome dyads from combined H4S47C- and H3Q85C-anchored cleavage data. Gene promoters aligned at +1 nucleosomes. Divergent (*top*) and tandem (*bottom*) genes are separated and sorted according to the distance between the +1 nucleosome and the NDR of the upstream gene. The *vertical red stripes* from the gene bodies indicate strong nucleosome phasing relative to the NDR. The nucleosome distribution in the region between neighboring NDRs is characterized by an interference pattern generated by phasing signals originating from both flanking NDRs. **b** Nucleosome distribution at the 3’ gene ends aligned at the transcription termination site (TTS). Convergent (*top*) and tandem (*bottom*) genes are separated and sorted according to the distance between the TTS and the NDR of the downstream gene. The absence of vertical red stripes at the TTS of convergent genes indicates that the TTS is not a nucleosome phasing element, unless it overlaps with the TSS of the downstream gene, as in the case of some of the tandem genes. Even in the case of tandem genes, it is evident that nucleosomes are not phased relative to the TTS but relative to the position of the nearby TSSs, as the red stripes are not vertical but bent according to the positions of the downstream genes. **c**, **d** Organization of in vitro reconstituted nucleosomes (data from [13]) near gene ends shows that DNA sequence is not sufficient to dictate nucleosome phasing patterns. In vitro, NDRs are formed on the regions of high A/T content, suggesting that they may be artifacts of MNase-seq, introduced by the MNase sensitivity of the nucleosomes located on A/T-rich sequences [68, 69], which are easily overdigested. **e**, **f** A/T content (ratio of nucleotides A or T) near gene ends shows that TTSs are among the most A/T-rich regions in the yeast genome. In vitro, NDRs near TTSs form at A/T-rich regions and not at the positions where they are observed in live cells

To better understand the nucleosome phasing patterns near gene promoters, we realigned all promoters at the +1 nucleosomes, and sorted the genes according to the distance between the +1 nucleosome and the NDR of the nearest upstream gene (Fig. 4a). We also separated the divergent genes (top part of Fig. 4a) from the tandem genes (bottom part of Fig. 4a). To maximize the signal, we combined dyads from our previously published H4S47C cleavage data (~ 300 million mapped nucleosomes, [26]) with the current H3Q85C cleavage data (~ 10 million mapped nucleosomes). The nucleosomes on the gene bodies are well phased relative to the NDR, as indicated by the regular pattern of vertical red stripes in Fig. 4a. The nucleosomes form regular arrays on both sides of the NDRs. Between neighboring NDRs, the nucleosome distribution produces an interference pattern, in which overlapping signals originate from each NDR [58]. When the space between the neighboring NDRs is a multiple of the typical nucleosome repeat length (NRL) (~ 165 bp in yeast [60]), we observe constructive interference of the two phasing signals, and well-positioned nucleosomes form a long regular array from one NDR to another. From Fig. 4a, we observe that nucleosome organization near gene promoters depends on the location and orientation of the upstream genes, and so averaging all configurations into an aggregate plot as is commonly done can be misleading, especially for the region upstream of the TSS.

We performed a similar analysis of the 3’ ends of the genes. It is known that the transcription termination site (TTS) is also a region of reduced nucleosome density, so the TTS could be another nucleosome phasing element, similar to the TSS. To test this hypothesis, we realigned all yeast genes at their TTSs and sorted according to the distance between the TTS and the NDR of the downstream gene (Fig. 4b). To distinguish the role of TTS *per se*, we separated the convergent genes (top part of Fig. 4b) from the tandem genes (bottom part of Fig. 4b), which typically have their TTS very close to the TSS of the neighboring downstream gene. We observed that the TTS of convergent genes is not characterized by strong nucleosome depletion. For tandem genes, strong nucleosome depletion is observed only when the TSS of the downstream gene is very close to the TTS of the reference gene. Moreover, the TTS is not a phasing element, and the only nucleosome phasing observed near the TTS is due to the presence of other TSSs in their vicinity. In the case of convergent genes, which have their TTS far from interfering TSSs of downstream genes, we do not observe vertical red stripes, which would indicate regular arrays of nucleosomes, phased relative to the TTS.

The presence of regular nucleosome arrays flanking the NDRs raises some interesting questions. Is this nucleosome organization encoded in the DNA sequence? Is the DNA sequence the most important factor that dictates the nucleosome positions in vivo? In order to answer these questions, we re-analyzed the nucleosome organization obtained by in vitro reconstitution [13]. Aligning the genes as in Fig. 4a, b, we see that reconstituted nucleosomes do not form the phased regular arrays flanking the NDRs (Fig. 4c, d), as observed in vivo (Fig. 4a, b). Instead, the nucleosome distribution is much more disorganized in vitro. Weak NDRs were observed in the reconstituted chromatin at both gene ends, with a stronger depletion present at the TTS. Surprisingly, strong NDR positions are observed for aligned TTSs in vitro, even though no strong NDRs are present in vivo. The locations of these strong in vitro NDRs coincide with the A/T-rich regions (Fig. 4e, f — genes aligned as in panels a, c and b, d, respectively). Although it is possible that there is reduced nucleosome formation at A/T-rich regions, the well-known preference of MNase for A/T-rich regions is sufficient to account for the NDRs at TTSs (Additional file 1: Figure S2) [20, 67]. In vitro, nucleosomes would form with similar affinities on every DNA sequence, but during the mapping process involving chromatin digestion by MNase, the nucleosomes from A/T-rich regions are digested faster, and are underrepresented in the sequenced library after extensive digestion (Additional file 1: Figure S2). In vivo, MNase sensitivity of nucleosomes wrapped by A/T-rich DNA sequences was previously reported both in *S. cerevisiae* [68] and in *Drosophila* [69].

### H3Q85C cleavage mapping demonstrates linker “quantization”

**Fig. 5:**
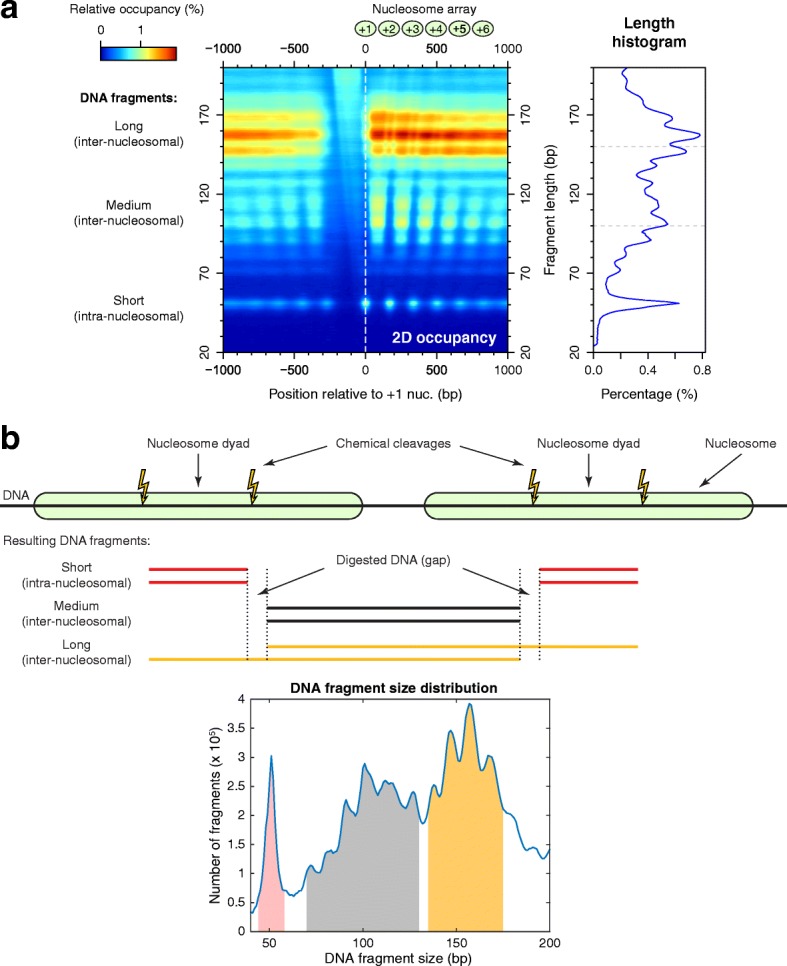
H3Q85C cleavage data prove linker “quantization.” **a** A two-dimensional occupancy heat map and length histogram show the coverage of DNA fragments of different sizes relative to +1 nucleosomes. There are three major groups of fragment sizes. Small fragments (~ 50 bp long) originate from two cuts inside the same nucleosome; their centers indicate the position of a nucleosome dyad. Medium fragments (~ 70–130 bp long) and long fragments (~ 135–175 bp long) originate from two cuts inside neighboring nucleosomes, produced at consecutive or alternate cleavage sites, respectively. **b** Scheme of the cleavage sites and the possible resulting fragments, together with the histogram of DNA fragment lengths obtained in the experiment

The precise measurements of dyad positions and nucleosome occupancy obtained using H3Q85C cleavage mapping also provide a rigorous method for investigating linker lengths and nucleosome crowding in vivo [70]. We addressed these issues using our full cleavage data sets, which were derived from DNA samples that had not undergone gel purification and so included dyad-to-dyad fragments spanning all linker lengths. We aligned the positions of all +1 nucleosomes and computed the relative occupancy generated by the DNA fragments of each different size (Fig. 5a). The short fragments (~ 50 bp) were concentrated around the locations of nucleosome dyads (Fig. 5a), and they originate from two cleavages inside the same nucleosome (red segments in Fig. 5b). The medium fragments (~ 70–130 bp) were located between the consecutive nucleosome dyad positions (Fig. 5a), which indicates that they were the fragments resulting from two cuts inside neighboring nucleosomes produced at consecutive cleavage sites (black segments in Fig. 5b). The long fragments (~ 135–175 bp) overlap both with short and medium fragments (Fig. 5a), because they derive from cuts made by different nucleosomes at alternate cleavage sites (yellow segments in Fig. 5b).

During the cleavage reaction and library preparation steps, the ends of the DNA fragments resulting from a cut are slightly shortened, and a small gap arises at the location of the cleavage site (Fig. 5b). In order to estimate the sizes of these gaps, we computed the cross-correlations between the ends of different groups of DNA fragments. To obtain the left gap between the short fragments and the medium or long fragments, we computed the cross-correlations between the right ends of the short fragments (44–58 bp) and the left ends of the medium fragments (130–170 bp) or the left ends of the long fragments (135–175 bp), respectively. The overall maxima of the cross-correlations correspond to a lag of 5 bp, which is equivalent to a gap of 4 bp between the fragments (Additional file 1: Figure S3). The same value was obtained for the second gap from Fig. 5, by calculating the cross-correlations between the left ends of the short fragments and the left ends of the medium or long fragments. This gap is expected between two cleavages by a single cysteine-phenanthroline on opposite strands 5 bp apart according to our published structural model [26].

Knowing the lengths of the sequenced fragments and the gaps between them, we next estimated the typical NRLs and linker lengths in *S. cerevisiae*. From the histogram of the DNA fragment lengths (Fig. 5b) we inferred that the most abundant lengths of the long fragments are 147 bp, 157 bp, and 167 bp. From Fig. 5b we see that the NRL can be computed as the sum of the gap size and the length of a long fragment, so we obtain for the most frequent NRLs the following values: 151 bp, 161 bp, and 171 bp. As the NRL is the sum of the length of the nucleosomal DNA fragment and the linker length, and nucleosomal DNA is ~ 146–147 bp long [1, 2], we conclude that the most frequent linker lengths in budding yeast are 5, 15, 25 or 4, 14, 24, depending on the exact value for the length of the nucleosomal DNA. This proves that the linker lengths are “quantized,” in the sense that the most favored values for the linker lengths are discretized and separated by 10 bp.

### Linker quantization at the gene level

The discretization of the linker lengths according to the rule *L = 10 n + 5*, at the global level, poses the following questions: Do some genes have linkers of 5 bp, others 15 bp, and others 25 bp? Or do individual genes also have a wide range of linker lengths, similar to the genome-wide linker length distribution? Are the linker lengths of an individual gene also discretized? Fortunately, our H3Q85C cleavage data permit the investigation of all these questions, as we can precisely map each internucleosomal DNA fragment to the correct location on the genome.

Yeast genes are often very small and are typically wrapped by only a few nucleosomes. In such cases, the number of reads mapped to the gene body is too low for reliably estimating linker sizes. Nevertheless, there are many yeast genes for which we obtained thousands of sequencing reads that allowed us to accurately estimate the linker length distribution for these genes.

**Fig. 6:**
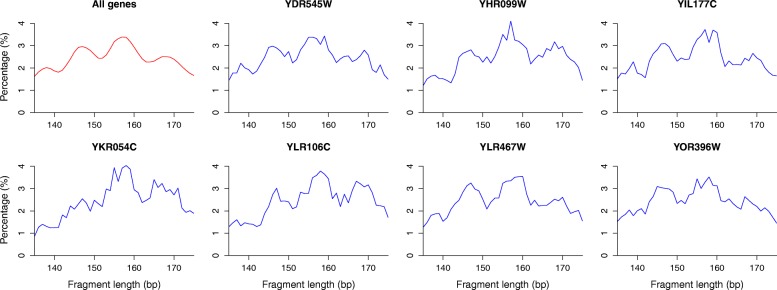
Nucleosome spacing and linker lengths are “quantized” even at the gene level. Length distribution of the long DNA fragments (135–175 bp) resulting from H3Q85C cleavages. Examples of yeast genes among the ones containing the highest numbers of reads over their gene bodies show that the distributions of fragment lengths from individual genes (*blue curves*) resemble the genome-wide distribution (*red curve*). This indicates that the same gene can have different levels of nucleosome compaction in different cells and at different times

We also analyzed the lengths of the long fragments (135–175 bp) that map to the bodies of single genes with > 5000 sequenced fragments (Fig. 6). We observed that individual genes are also characterized by a wide range of fragment lengths, similar to the genome-wide distribution (red curve in Fig. 6). This shows that in different cells and at different times during the cell cycle, individual genes can have somewhat different nucleosome organizations and NRLs, suggesting that chromatin organization is reacting to the stochastic processes that take place inside the nuclei.

### Correlations among nucleosome spacing, transcription rate, and NDR width

**Fig. 7:**
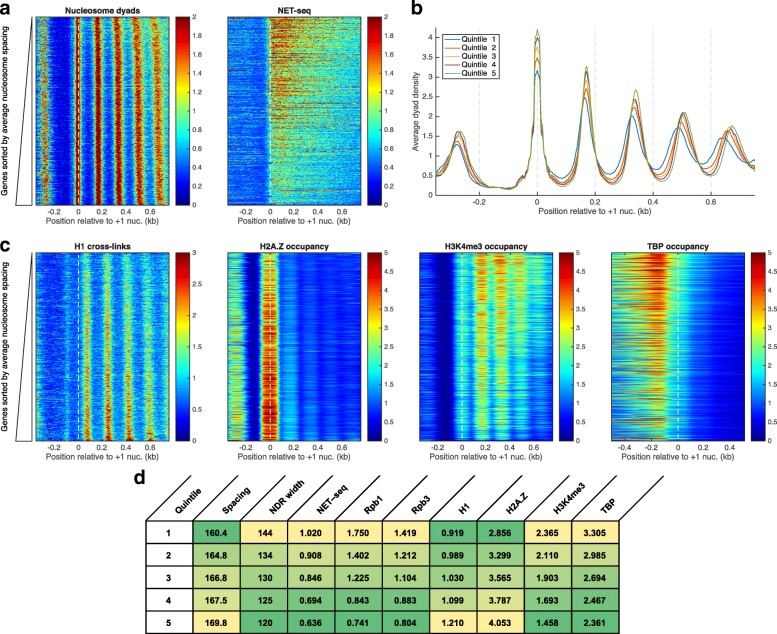
Genes sorted by average size of the long fragments from H4Q85C cleavage data. **a** Genes aligned at the +1 nucleosome and sorted according to the average size of the long fragments (135–175 bp) that were produced by H3Q85C cleavage. The transcription levels, as measured by the levels of nascent transcripts obtained from NET-seq data [73], indicate that the more transcribed genes have more compact nucleosome arrays. **b** Average nucleosome dyad density for the yeast genes separated into five quintiles: quintile 1, the top fifth from **a**, through quintile 5, the bottom fifth from **a**. The decrease in nucleosome spacing correlates with a widening of the NDR, which is characteristic for the highly transcribed genes. **c** The distribution of linker histone H1 (Hho1 data from [80]) is also correlated with nucleosome spacing: the longer the nucleosome spacing, the more H1 is bound to the corresponding nucleosomes. The H2A.Z variant is enriched in the +1 nucleosomes (data from [61]); the less active genes appear to have more H2A.Z histones incorporated into their +1 nucleosomes. The more active genes contain more H3K4me3 marks on their bodies (data from [81]) and more TBP bound to their promoters (data from [62]). **d** Average properties for the five quintiles

One of the factors shown to have an effect on the nucleosome spacing is the level of transcription of a gene [71, 72]. In general, highly transcribed genes are characterized by a decreased nucleosome spacing [72], and the inactivation of RNA polymerase II via a temperature-sensitive mutation was shown to increase the nucleosome spacing [71]. As previously noted, the nucleosome phasing pattern can be explained to a first approximation by statistical positioning against promoter barriers [46]. The number of barrier complexes that are bound to a promoter is presumably correlated with the width of the accessible region, the NDR width. To test for possible relationships between NDR widths, transcription rates, and nucleosome spacing, we computed for each gene the average size of the long fragments (135–175 bp) from H3Q85C cleavage data, and we re-sorted the yeast genes according to this score (Fig. 7a; the genes with the longest average length of the H3Q85C cleavage fragments are at the bottom). This sorting score is a direct measure of the average nucleosome spacing, and it is closely related to the average linker length for each gene. As expected, the heat map confirmed that the arrays of nucleosomes at the top are more crowded, while the nucleosome arrays at the bottom are less crowded (toward the bottom of the heat map, the red stripes indicating nucleosome dyads bend to the right). This increase in nucleosome spacing was also observed when we grouped the genes from the heat map into five groups: quintile 1 containing the top fifth of the rows, quintile 5 containing the bottom fifth of the rows, etc. (Fig. 7b). The first quintile, with the most crowded nucleosomes, also had the widest NDR (Fig. 7b), which is characteristic of highly transcribed genes [51, 71]. To further test for a correlation between the chromatin organization and the transcription levels, we analyzed the levels of nascent transcripts obtained from native elongating transcript sequencing (NET-seq) data [73]. Indeed, transcription levels correlate with chromatin organization; the more active genes are the ones at the top of the heat map, corresponding to the genes with more crowded nucleosome arrays and wider NDRs (Fig. 7a).

Chromatin organization and nucleosome spacing also correlate with the amounts of linker histone H1 (Hho1 in yeast) present on the gene bodies and the level of incorporation of the H2A.Z variant into the +1 nucleosomes. Linker histone H1 is preferentially deposited on the genes with increased nucleosome spacing (Fig. 7c), in agreement with observations from multicellular organisms, where histone H1 and its variants have an important role in nucleosome spacing [74, 75]. Moreover, genes with increased nucleosome spacing also contain the +1 nucleosomes with the highest incorporation of H2A.Z. This agrees with the previous observation that, in yeast, H2A.Z is localized to the promoters of less active genes [76, 77], suggesting that during transcription, histone variant H2A.Z is preferentially evicted compared to the canonical histone H2A. As the genes with the more crowded nucleosome arrays from the top of the heat map are more transcribed, their gene bodies are marked by characteristic histone modifications, such as H3K4me3 (Fig. 7c), and their promoters contain higher amounts of TBP, which is probably an underlying cause of the extended NDRs of these genes. For each quintile shown in Fig. 7b, we computed the following quantities (Fig. 7d): average nucleosome spacing and average NDR width, estimated from Fig. 7b; average NET-seq signal on the gene bodies (data from [73]); average Rpb1 density on the gene bodies (data from [78]); average Rpb3 density on the gene bodies (data from [79]); average H1 cross-link density in the window (D-73, D + 750) bp, where D is the dyad position for +1 nucleosome (data from [80]); average H2A.Z occupancy for the +1 nucleosomes (data from [61]); average H3K4me3 occupancy computed in the same range as for the H1 density (data from [81]); average TBP occupancy in the window (D-500, D-73) bp, where D is the dyad position for +1 nucleosome (data from [62]). The strong positive and negative correlations suggested by the heat maps in Fig. 7 are confirmed for all possible comparisons by Pearson correlation coefficients and scatter plots (Additional file 1: Figure S4). In particular, the five quintiles show near-perfect correlations between the average NDR width, transcription level (estimated by the average NET-seq, Rpb1, and Rpb3 signals on the gene bodies, *r* = 0.980–0.988), average TBP occupancy at promoters (*r* = 0.988), and average density of H3K4me3 marks (*r* = 0.989). All these quantities are almost perfectly anti-correlated with the average nucleosome spacing of the corresponding genes, average enrichment of histone variant H2A.Z in the +1 nucleosomes, and with the average density of linker histone H1 (*r* = –0.945 to 0.998).

### A biophysical model for nucleosome positioning

**Fig. 8:**
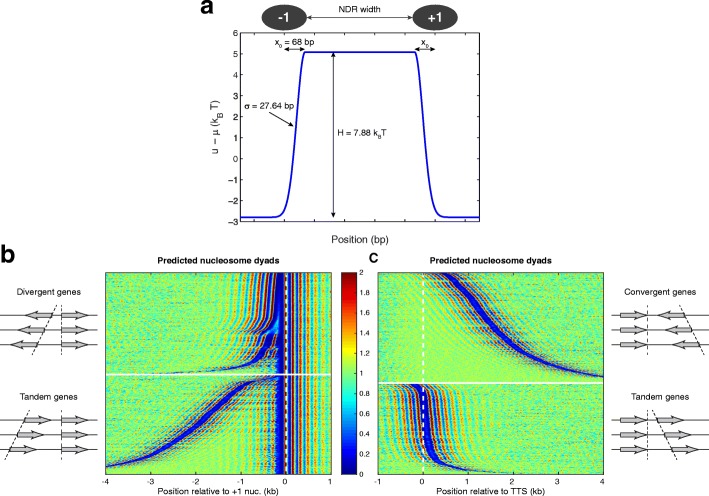
Nucleosome organization modeled by statistical mechanics predicts the correct phasing pattern and NDR locations. **a** Fitted parameters for the energy barrier localized at the gene promoters. The barrier consists of two halves of a Gaussian distribution (*H* = 7.88 *k*_B_*T*; σ = 27.64 bp) with the centers shifted by *x*_0_ = 68 bp from the dyads of +1/–1 nucleosomes and a flat energy barrier in between. The additional fitting parameter, the binding energy for a nucleosome away from the barrier, *u* − *μ* = –2.79 *k*_B_*T*. **b** Predicted nucleosome organization in gene promoters. Promoters are aligned at +1 nucleosomes and sorted exactly as in Fig. 4a. **c** Predicted nucleosome distribution at the 3’ ends of genes, which are aligned at the TTS. Genes are sorted as in Fig. 4b. A discussion of the applications of Tonks gas modeling of nucleosome positions is presented elsewhere [105]

In general, promoters are depleted of nucleosomes and are occupied by other non-histone protein complexes (Fig. 3). This competition among diverse proteins for binding to the same DNA can be modeled using statistical mechanics methods [46, 69, 70]. Briefly, nucleosomes are modeled as a system of non-overlapping one-dimensional (1D) particles of length *a* = 147 bp. The particles are confined to 1D lattices of different lengths, representing the 16 yeast chromosomes. The energy of the system is given by the sum of one-particle energies (the binding energies corresponding to each nucleosome, *u*(*i*), where *i* represents the coordinate of the corresponding nucleosome dyad) and the two-body interactions, Φ(*i*, *j*), where *i* and *j* represent the positions of the neighboring nucleosome dyads. We assume a hard-core interaction between the nucleosomes (infinite if nucleosomes overlap, and zero if they do not overlap) which guarantees that only the valid non-overlapping nucleosomal configurations have finite energies and non-vanishing probabilities. This is also known as a Tonks lattice gas model [82]. The effect of non-histone barrier complexes that bind to promoters and create the NDRs is modeled by an external energy barrier that increases the binding energy of nucleosomes located in the promoters, preventing nucleosome formation at these loci. Following the formalism described in [69] and illustrated in Additional file 1: Figure S5, we enumerate all valid nucleosome configurations and their corresponding energies and probabilities in a recursive way. Once we compute the normalization factor of the Boltzmann weights corresponding to each state (the grand canonical partition function, *Z*), we predict the distribution of nucleosome dyads and the nucleosome occupancy by summing the probabilities of all states that contain a nucleosome at a given position, or a nucleosome covering a specified base pair, respectively. Using the nucleosome distribution on chromosome I for training the model, we calculated the model parameters — the chemical potential, *μ*, related to the overall nucleosome density, the average nucleosome binding energy, *u*, and the energy barrier parameters, *H*, *σ*, *x*_0_ (Fig. 8a) — such that the predicted nucleosome organization on chromosome I fits the data. The fitted parameters were then used to predict the genome-wide nucleosome organization.

Figure 8b shows the predicted nucleosome distribution at the gene ends, which are aligned as in Fig. 4. In this model the effect of the entire environment upon nucleosome formation is reduced to the presence of simple energy barriers at promoters, and the sequence-dependent contribution to nucleosome affinity is completely neglected. It is striking that the model produces a close approximation of the nucleosome phasing pattern. Although the energy barriers are placed only at the 5’ ends of the genes, they are enough to also generate the correct organization at the 3’ ends of the genes (Fig. 8c), which supports the hypothesis that TTSs are not nucleosome phasing elements. Therefore, the nucleosome organization at the 3’ ends of genes is also dictated by phasing elements at the 5’ ends of genes.

In vivo, there are also other processes that cannot be easily modeled within the framework of statistical positioning models. Transcription is a major nucleosome disrupting process [49–51, 83], and chromatin remodelers were shown to have a major impact on nucleosome organization [56, 58–60]. In order to model these processes, non-equilibrium statistical mechanics models are necessary, but single-molecule experiments that could be used to train such models are not yet available.

## Discussion

We have developed an improved in vivo chemical cleavage method that allows simultaneous precise mapping of both nucleosomes and DNA linkers. Our H3Q85C cleavage method determines individual nucleosome positions with high accuracy, while avoiding complications due to differential MNase sensitivity of nucleosomes [67]. Unlike the earlier H4S47C cleavage method, H3Q85C cleavage maps individual nucleosomes, which avoids phenanthroline-mediated linker noise and greatly increases coverage, resulting in robust detection of rotational phasing. Our method also detects pairs of neighboring nucleosomes from the same cell without relying on any statistical inference model — each sequencing read precisely determines either the position of a single nucleosome or the spacing between two neighboring nucleosomes. As opposed to the rough estimations of the overall NRL that one could obtain from gel electrophoresis of MNase-digested chromatin [84], our method offers high-resolution measurements of the NRL at the single-gene level, and even at the single-linker level, using increased sequencing depth.

In addition to providing improved nucleosome maps, our method can address outstanding biological questions that could not be resolved using previous methods. Our H3Q85C maps revealed the absence of histone H3 at yeast centromeres, ruling out a model that proposed a Cse4-H3 heterotypic nucleosome [33] at each yeast centromere. We also showed that tRNA genes are depleted of nucleosomes, which could not be determined using the H4S47C method, presumably because of phenanthroline-mediated background at NDRs. Single nucleosome mapping also led to the discovery of three alternative rotational positions on either side of the central dyad position and rotational positioning of nucleosomes remodeled by RSC, demonstrating that variation in the position of the dyad follows integral multiples of helical twist. Our results suggest that at the single-nucleosome level, the histone core is able to sample an ensemble of favorable rotational positions determined by the underlying DNA sequence, and that chromatin remodelers affect the stochastic process of selecting the individual positions of nucleosomes in each cell.

Given the high resolution of our measurements, we have been able to show that linkers between neighboring nucleosomes have a wide distribution of lengths, with preferred discrete lengths according to the rule *L = 10 n + 5*, where *n* is an integer [85]. Linker discretization was previously inferred from analysis of dinucleotide periodicities of 296 mononucleosomal DNA sequences [86], but direct measurements of dinucleosomal data at the genome-wide scale are not yet available to confirm this linker discretization rule. The same linker discretization rule was suggested in more recent studies [23, 27], where this rule was obtained from the “unique nucleosome positions” defined by clustering the chemical cleavages and inferring the most frequent positions among a population of cells. Again, dinucleosomal fragments originating from the same cell were not used to test this hypothesis. When the paired-end H4S47C cleavage data [23] were re-analyzed without neglecting the joint distribution of the pairs of neighboring cleavages originating from the same cells [70], a linker discretization rule was not found. Nucleosomal dimer fragments from rat liver chromatin, obtained by MNase digestion combined with exonuclease III and S1 nuclease trimming, were also used to suggest that the lengths of internucleosomal DNA are integral multiples of the helical repeat plus 5 bp [87], although the oscillatory signal obtained in the distribution of dinucleosomal fragment lengths was weak [87] and even absent in other virtually identical studies [88, 89].

Our H3Q85C cleavage method allows precise measurements both of individual nucleosome positions and individual linker lengths between neighboring nucleosomes originating from the same cell. We have shown that the linker discretization rule holds not only at the genome-wide level, but also at the single-gene level, indicating significant heterogeneity of nucleosome configurations on the coding regions within a cell population. A similar high variability in nucleosome positions among individual cells was previously reported for the *PHO5* promoter, the 5’ end of the *CHA1* gene, and the promoter of *CYS3* [32].

It is known that chromatin packaging in different organisms is characterized by a variety of NRLs, ranging from 154 bp in *Aspergillus nidulans* [90] to 260 in *Strongylocentrotus purpuratus* sperm [91] (for an extended list of NRLs in different organisms see [85, 92]). Our data indicate that even individual genes of the same organism may have a wide range of different NRLs among a population of cells. This suggests that in different cells the same gene may have different chromatin conformations, depending on the corresponding linker lengths [92, 93].

Using the precise measurements of linker lengths at the single-gene level, we were able to rank yeast genes according to the typical degree of nucleosome crowding, which allowed the further investigation of the role of nucleosome spacing in other biological processes. As a demonstration of the validity of the method, we recapitulated the well-known correlations between nucleosome spacing and multiple chromatin properties: depletion of nucleosomes from promoters, incorporation of histone variant H2A.Z in the +1 nucleosome and of linker histone H1 in the coding regions, and transcription level. We confirmed that the most transcribed genes have a decreased nucleosome spacing and a larger NDR width [71, 72]. This indicates that during transcription initiation promoters of highly transcribed genes are occupied by other protein complexes that compete with nucleosome formation, increasing the NDR width.

We have shown that a simple biophysical model can explain the formation of NDRs in promoters and the nucleosome phasing patterns observed near both gene ends. We have used statistical mechanics to model the distribution of hard rods, representing nucleosomes, in 1D lattices, representing chromosomes [46, 94]. As opposed to previous genome-wide nucleosome predictions [38, 94], we designed a minimal model that does not rely on fitting sequence-dependent nucleosome affinities, but uses the steric exclusion principle as the only requirement, in order to explain the nucleosome phasing near external energy barriers located in the gene promoters (representing the competition with other DNA-binding proteins that are found in promoters). This Tonks gas system was previously used to explain the oscillatory nucleosome distribution near a perfect barrier [44] and near TSSs [95]. By fitting the energy barrier from promoters, we extend the prediction of nucleosome positions genome-wide and show that the complex nucleosome phasing pattern can be explained at the genome-wide scale, including the 3’ ends of genes, where no phasing element was imposed. Using the smallest yeast chromosome alone, we trained our model and inferred the energy barriers created by other DNA-binding proteins, which compete with histones and prevent nucleosome formation in the NDRs. Our genome-wide prediction of nucleosome organization is in excellent agreement with the experimental data, indicating that barrier complexes that occupy promoters are essential for establishing the nucleosome organization in vivo, and our minimal biophysical model is able to explain the pattern of nucleosome phasing genome-wide.

Our biophysical model explains the salient features of nucleosome positioning genome-wide, while ignoring DNA sequence, thus simplifying our current understanding of the rules for nucleosome positioning [15]. Our model also can account for previous observations that DNA sequence is unable to generate the stereotypical nucleosome organization near TSSs, without help from ATP-dependent chromatin remodelers [57, 96]. Further improvements in the prediction of nucleosome positions might be obtained by including in the model additional energy barriers at other NDRs, such as those observed at tRNA genes [97] and origins of replication [98].

## Conclusions

Our introduction of a new method for mapping nucleosomes that is not subject to the uncertainties inherent in MNase-seq and H4S47C cleavage mapping has led to new insights into the forces governing nucleosome positioning. Our approach provides high-confidence validation of relationships between nucleosome spacing, NDR width, transcription, and chromatin features genome-wide. We have shown that rotational positioning is simply described as A/T out of phase with G/C, that TTSs are not positioning elements, and that linkers are quantized on the level of a gene. Our minimal biophysical model, which neglects sequence-dependent nucleosome affinities, can explain the formation of NDRs and nucleosome phasing patterns and implies that steric exclusion plays a central role in establishing nucleosome phasing.

## Methods

### H3Q85C cleavage mapping

#### Strain construction

Point mutations in histone H3 were introduced into the HHT2/HHF2 plasmid, which contains a *URA3* selectable marker [99]. The locus with homology arms to the genomic HHT2/HHF2 locus was excised together with *URA3* from the mutant plasmid using *Bci*IV and transformed into the W1588-4C strain with the HHT1/HHF1 locus deleted (*MATa ade2-1 can1-100 his3-11, 15 leu2-3, 112 trp1-1 ura3-1 RAD5+ -* Δhht1/hhf1::*kanMX4*). Transformed cells were selected for on minimal medium-uracil plates to obtain cells with single copies of the histone H3/H4 genes with the required mutations in histone H3.

#### Cleavage mapping experiments

The yeast strain with HHT1/HHF1 deleted and carrying the H3Q85C mutation was grown in a volume of ~ 166 ml in yeast extract peptone dextrose (YPD) medium up to optical density (OD)_600_ = 0.4–0.6. Cells were collected by centrifugation at 3000 g for 5 min. Cells were resuspended with 1 ml of 1 M sorbitol and spun again at 3000 g for 5 min. Cells were resuspended with 1 ml of spheroplasting buffer containing lyticase (1 M sorbitol, 5 mM β-mercaptoethanol, 10 mM Tris, 10 mg/ml lyticase) and gently rotated at room temperature for 5 min. Cells were pelleted at 3000 g for 5 min and washed twice with 1.5 ml of sorbitol/NP-40 buffer (1 M sorbitol, 0.2% NP-40 (# 74385, Sigma-Aldrich, St. Louis, MO, USA), and 10 mM Tris pH 7.5). The cell pellet was resuspended in 400 μl of labeling buffer (1 M sorbitol, 50 mM NaCl, 10 mM Tris-HCl pH 7.5, 5 mM MgCl_2_, 0.5 mM spermidine, 0.15 mM spermine, 0.2% NP-40, 0.1 mM ethylenediaminetetraacetic acid (EDTA)). At this point, the tubes were covered with aluminum foil to protect them from light. The phenanthroline label, *N*-(1,10-phenanthroline-5-yl)iodoacetamide (# 92015, Biotium, Fremont, CA, USA) stock solution was made fresh by dissolving the lyophilized label to a concentration of 7 mM in dimethyl sulfoxide (DMSO). The label was added to cells for a final concentration of 0.14 mM. The labeling mixture was rotated at room temperature for 2 h and then at 4 °C overnight. The cells were washed three times in 1.5 ml of sorbitol/NP-40 buffer to remove excess label. Comparison of cells before and after phenanthroline labeling by MNase-seq showed no evidence of changes in nucleosome position or occupancy [26].

Phenanthroline-labeled cells were resuspended in 300 μl of mapping buffer (2.5 mM NaCl, 50 mM Tris-HCl pH 7.5, 5 mM MgCl_2_, 1 M sorbitol, 0.5 mM spermidine, 0.15 mM spermine, 0.2% NP-40), CuCl_2_ was added to a final concentration of 150 μM, and the cells were incubated at room temperature for 2 min. Cells were spun down for 3 min at 3400 g and then washed three times with 1.5 ml of mapping buffer. After the final wash, the cells were resuspended in 300 μl of mapping buffer. 3-Mercaptopropionic acid was then added to a final concentration of 6 mM followed by H_2_O_2_ to a final concentration of 6 mM. The reaction was incubated at room temperature for 15 min, quenched by the addition of neocuproine to 0.28 mM, and the cells were spun down at 3000 g for 5 min. The reaction was performed four more times starting with resuspension in mapping buffer. The cells were pelleted after five reactions and resuspended in 400 μl of Tris-EDTA (TE) buffer (10 mM Tris-HCl pH 8.0, 1 mM EDTA). EDTA was supplemented to a final concentration of 10 mM and sodium dodecyl sulfate was added to a final concentration of 0.5%. Twenty μg of proteinase K was added, and the samples were incubated at 55 °C for 2 h. To extract DNA, two volumes (800 μl) of phenol:chloroform:isoamyl alcohol (PCI, 25:24:1) was added to the samples and mixed well, and the samples were spun at max speed for 5 min. The aqueous phase was separated and the DNA precipitated with three volumes of 100% ethanol and 30 μg of GlycoBlue as a carrier, for 30 min at 4^o^ C, maximum speed. DNA pellets were washed with 1 ml of 75% ethanol and spun again. The pellets were air dried and resuspended in 50–100 μl of TE buffer. Then 90 μg of RNase A was added and the samples incubated at 42 °C for 30 min. We analyzed 2 μg of cleavage material and 0.5 μg of starting material by PAGE (10% acrylamide). If the 50-bp band was observed, a final PCI extraction, chloroform cleanup, and ethanol precipitation were performed before proceeding to library preparation. For one of the samples, the 50-bp fragment band was excised from a polyacrylamide gel and purified before making libraries.

### Sequencing

Cluster generation and 25 rounds of paired-end sequencing were performed by the Fred Hutchinson Cancer Research Center (FHCRC) Genomics Shared Resource with an Illumina Hi-Seq 2500. Base calling, data processing, and analysis were performed as described [18].

### Cleavage data analysis

Paired-end reads were aligned to the *S. cerevisiae* reference genome *sacCer3*, using Bowtie2 [100] with parameters *-X 1000 --very-sensitive*, to map sequences up to 1 kb with maximum accuracy. DNA fragments of lengths 51 ± 7 bp were selected and their centers used to define the nucleosome dyads. To estimate the nucleosome occupancy, each dyad was extended 50 bp in both directions, resulting in a footprint of 101 bp for each nucleosome. The occupancy profiles obtained by stacking the reduced footprints (< 147 bp) are useful for emphasizing the linkers between neighboring nucleosomes when they occupy alternative positions in different cells. The detection of NDRs and flanking (+1/–1) nucleosome positions was done as in [60]. Heat maps representing different genomic data, aligned at various references (TSS, TTS, and NDR center) were generated in MATLAB using the *Bioinformatics* toolbox to import data and the *heatmap* plotting function (http://www.mathworks.com/matlabcentral/fileexchange/24253-customizable-heat-maps). To visualize specific loci, the Integrative Genomics Viewer (IGV) browser [101] was used, loading the tracks (tdf files) created with *igvtools*. Transcript end coordinates (TSS and TTS) were obtained from a previous work [102].

### Precise identification of the +1 and –1 nucleosomes

Using a previously described procedure [60], we first identified all NDRs and the typical locations of +1 and –1 nucleosomes in *S. cerevisiae*. Nucleosomes do not occupy identical positions in all cells, and for each locus occupied by nucleosomes we observed clusters of alternative rotational positions in the ensemble of cells. To identify the center of the cluster of alternative positions, we searched in a window of 50 bp around the previously identified positions, and we refined the positions of the +1 and –1 nucleosomes by maximizing the cross-correlation between the cumulative profile of dyad counts and the following oscillatory pattern:

$$ {\displaystyle \begin{array}{l}{D}_0(x)=G\left(x,0,2\right)+0.75\ G\left(x,10,2\right)+0.50\ G\left(x,20,2\right)+0.25\ G\left(x,30,2\right)\\ {}+0.75\ G\left(x,-10,2\right)+0.50\ G\left(x,-20,2\right)+0.25\ G\left(x,-30,2\right),\end{array}} $$

where $G\left(x,c,\sigma \right)={e}^{\frac{-{\left(x-c\right)}^2}{2\ {\sigma}^2}}$ represents a Gaussian distribution with mean *c* and standard deviation *σ*. The cross-correlations were computed using the *xcorr* function from the *Signal Processing* toolbox of MATLAB.

### Two-dimensional occupancy analysis

To study the origin of the fragments of different sizes that resulted in the chemical cleavage reaction, we used two-dimensional (2D) occupancy analysis [68]. Briefly, for each fragment size, we computed the relative occupancy due to all DNA fragments of this size. Then we created a matrix with the rows representing the relative occupancy corresponding to each DNA fragment size, and the columns representing the number of fragments of different sizes covering each base pair in promoter regions. This 2D representation of occupancy (Fig. 5a) allowed us to distinguish fragments of different sizes and to identify their origin as a function of their lengths. The columns of the 2D occupancy profile (Fig. 5a) mimic the separation of DNA fragments in a gel electrophoresis experiment, while the rows provide information on their genomic locations. The marginal distribution obtained by summing all values from each column of this matrix will generate the usual 1D occupancy, while the marginal distribution obtained by summing all values from each row of this matrix will generate the DNA fragment length histogram (Fig. 5a, right panel).

The software used for computing and visualizing the 2D occupancies is freely available on the GitHub repository page at https://github.com/rchereji/plot2DO. For more information on usage options, consult the online documentation from the GitHub page.

### Analysis of the missing gaps between chemical cleavage fragments

Two neighboring DNA fragments result from each chemical cleavage. During the cleavage process or throughout the library preparation steps, a few nucleotides between the two resulting fragments are lost. The size of the gap is important in order to estimate the NRL, and we estimate this by computing the lag that maximizes the cross-correlation between the ends of the two sets of resulting DNA fragments: short fragments (51 ± 7 bp) originating from two cleavages in the same nucleosome, and longer fragments (70–130 bp) originating from two cleavages on distinct neighboring nucleosomes (Fig. 5b). The cross-correlations are computed using the *xcorr* function from the *Signal Processing* toolbox of MATLAB.

### Statistical mechanics model

The statistical mechanics formalism used to predict the nucleosome organization near the energy barriers located in the gene promoters is described in [69]. Briefly, a chromosome containing *L* bp is modeled as a 1D lattice of length *L*, which is populated by hard rods of size *a* = 147 bp. Each position *i* where a nucleosome can form along the chromosome is characterized by a binding energy *u*(*i*), which is constant everywhere along the chromosome, except for the positions at promoters, where the potential barriers dictate the binding energy. Assigning to each valid configuration (in which the hard rods do not overlap) a Boltzmann weight according to the total energy of the system, and taking into account all the possible configurations of the system with a variable number of hard rods, we compute the grand canonical partition function [103] as described in [69]. The partition function can be computed in a recursive way, and from this function we obtain the probability for a nucleosome dyad to occupy at position *i*, *n*(*i*), and the probability that base pair *i* is occupied by a nucleosome, or nucleosome occupancy:

$$ Occ(i)=\sum \limits_{k=i-73}^{i+73}n(k). $$

This way, knowing the potential barriers from promoters, we can predict the distribution of nucleosomes on the whole genome. We train the model by fitting the nucleosome distribution on the shortest yeast chromosome, and with the energy parameters that we obtained (Fig. 8a) we predict the genome-wide nucleosome organization (Fig. 8b, c). A generalized formalism, in which generic two-body interactions are considered between neighboring nucleosomes [46] or nucleosomes can be partially unwrapped [70], is also available.

### Model training

In order to train the model and to obtain the parameters shown in Fig. 8a, a global optimization was performed using the genetic algorithm optimization function *ga* from the *Global Optimization* toolbox of MATLAB. The residue function that was minimized by the global optimization function consisted of the negative of the 2D correlation coefficient (*corr2* function from the *Image Processing* toolbox in MATLAB) between the real and the predicted nucleosome dyad distributions on chromosome I, aligned at the +1 nucleosome (as in Fig. 8b, but using only the rows/genes corresponding to chromosome I).

## Additional files

Additional file 1: Figure S1. Chemical cleavages produced by H3Q85C. **Figure S2.** H3Q85C cleavage mapping distinguishes between real NDRs and MNase artifacts (MNase-sensitive nucleosomes). **Figure S3.** Determination of the gap size using cross-correlations between the distributions of the DNA fragment ends. **Figure S4.** Correlations among nucleosome spacing, NDR width, transcription levels, and ChIP-seq data. **Figure S5.** Sketch of the statistical mechanics formalism for predicting nucleosome occupancy. (PDF 2035 kb)

Additional file 2: Table S1 The coordinates of the dominant positions of +1 and –1 nucleosomes identified in this study. (XLSX 396 kb)

## Abbreviations

MNase: Micrococcal nuclease
MNase-seq: Micrococcal nuclease followed by high-throughput sequencing
NDR: Nucleosome-depleted region
NRL: Nucleosome repeat length
TSS: Transcription start site
TTS: Transcription termination site

## References

[1] Luger K, Mäder AW, Richmond RK, Sargent DF, Richmond TJ (1997). Crystal structure of the nucleosome core particle at 2.8 A resolution. Nature.

[2] Davey CA, Sargent DF, Luger K, Maeder AW, Richmond TJ (2002). Solvent mediated interactions in the structure of the nucleosome core particle at 1.9 a resolution. J Mol Biol.

[3] Flaus A, Luger K, Tan S, Richmond TJ (1996). Mapping nucleosome position at single base-pair resolution by using site-directed hydroxyl radicals. Proc Natl Acad Sci U S A..

[4] Lorch Y, LaPointe JW, Kornberg RD (1987). Nucleosomes inhibit the initiation of transcription but allow chain elongation with the displacement of histones. Cell..

[5] Han M, Grunstein M (1988). Nucleosome loss activates yeast downstream promoters in vivo. Cell..

[6] Li B, Carey M, Workman JL (2007). The role of chromatin during transcription. Cell..

[7] Bai L, Morozov AV (2010). Gene regulation by nucleosome positioning. Trends Genet..

[8] Groth A, Rocha W, Verreault A, Almouzni G (2007). Chromatin challenges during DNA replication and repair. Cell..

[9] Eaton ML, Galani K, Kang S, Bell SP, MacAlpine DM (2010). Conserved nucleosome positioning defines replication origins. Genes Dev..

[10] Adkins NL, Niu H, Sung P, Peterson CL (2013). Nucleosome dynamics regulates DNA processing. Nat Struct Mol Biol..

[11] Bevington S, Boyes J (2013). Transcription-coupled eviction of histones H2A/H2B governs V(D)J recombination. EMBO J..

[12] Pugh BF (2010). A preoccupied position on nucleosomes. Nat Struct Mol Biol..

[13] Kaplan N, Moore IK, Fondufe-Mittendorf Y, Gossett AJ, Tillo D, Field Y, LeProust EM, Hughes TR, Lieb JD, Widom J, Segal E (2009). The DNA-encoded nucleosome organization of a eukaryotic genome. Nature..

[14] Kaplan N, Moore I, Fondufe-Mittendorf Y, Gossett AJ, Tillo D, Field Y, Hughes TR, Lieb JD, Widom J, Segal E (2010). Nucleosome sequence preferences influence in vivo nucleosome organization. Nat Struct Mol Biol..

[15] Struhl K, Segal E (2013). Determinants of nucleosome positioning. Nat Struct Mol Biol..

[16] Zhang Y, Moqtaderi Z, Rattner BP, Euskirchen G, Snyder M, Kadonaga JT, Liu XS, Struhl K (2010). Evidence against a genomic code for nucleosome positioning. Reply to "Nucleosome sequence preferences influence in vivo nucleosome organization.". Nat Struct Mol Biol.

[17] Fan X, Moqtaderi Z, Jin Y, Zhang Y, Liu XS, Struhl K (2010). Nucleosome depletion at yeast terminators is not intrinsic and can occur by a transcriptional mechanism linked to 3'-end formation. Proc Natl Acad Sci U S A..

[18] Henikoff JG, Belsky JA, Krassovsky K, MacAlpine DM, Henikoff S (2011). Epigenome characterization at single base-pair resolution. Proc Natl Acad Sci U S A..

[19] Hörz W, Altenburger W (1981). Sequence specific cleavage of DNA by micrococcal nuclease. Nucleic Acids Res..

[20] Chung H-R, Dunkel I, Heise F, Linke C, Krobitsch S, Ehrenhofer-Murray AE, Sperling SR, Vingron M (2010). The effect of micrococcal nuclease digestion on nucleosome positioning data. Plos One..

[21] Dingwall C, Lomonossoff GP, Laskey RA (1981). High sequence specificity of micrococcal nuclease. Nucleic Acids Res..

[22] Mieczkowski J, Cook A, Bowman SK, Mueller B, Alver BH, Kundu S, Deaton AM, Urban JA, Larschan E, Park PJ (2016). MNase titration reveals differences between nucleosome occupancy and chromatin accessibility. Nat Commun..

[23] Brogaard K, Xi L, Wang J-P, Widom J (2012). A map of nucleosome positions in yeast at base-pair resolution. Nature..

[24] Statham AL, Taberlay PC, Kelly TK, Jones PA, Clark SJ (2015). Genome-wide nucleosome occupancy and DNA methylation profiling of four human cell lines. Genom Data..

[25] Flaus A, Richmond TJ (1999). Base-pair resolution mapping of nucleosomes in vitro. Methods Mol Biol..

[26] Henikoff S, Ramachandran S, Krassovsky K, Bryson TD, Codomo CA, Brogaard K, Widom J, Wang J-P, Henikoff JG (2014). The budding yeast Centromere DNA Element II wraps a stable Cse4 hemisome in either orientation in vivo. Elife..

[27] Moyle-Heyrman G, Zaichuk T, Xi L, Zhang Q, Uhlenbeck OC, Holmgren R, Widom J, Wang J-P (2013). Chemical map of Schizosaccharomyces pombe reveals species-specific features in nucleosome positioning. Proc Natl Acad Sci U S A..

[28] Thakur J, Talbert PB, Henikoff S (2015). Inner kinetochore protein interactions with regional centromeres of fission yeast. Genetics..

[29] Voong LN, Xi L, Sebeson AC, Xiong B, Wang J-P, Wang X (2016). Insights into nucleosome organization in mouse embryonic stem cells through chemical mapping. Cell.

[30] Fragoso G, John S, Roberts MS, Hager GL (1995). Nucleosome positioning on the MMTV LTR results from the frequency-biased occupancy of multiple frames. Genes Dev..

[31] Shen CH, Leblanc BP, Alfieri JA, Clark DJ (2001). Remodeling of yeast CUP1 chromatin involves activator-dependent repositioning of nucleosomes over the entire gene and flanking sequences. Mol Cell Biol..

[32] Small EC, Xi L, Wang JP, Widom J, Licht JD (2014). Single-cell nucleosome mapping reveals the molecular basis of gene expression heterogeneity. Proc Natl Acad Sci U S A..

[33] Lochmann B, Ivanov D (2012). Histone h3 localizes to the centromeric DNA in budding yeast. PLoS Genet..

[34] Shrader TE, Crothers DM (1989). Artificial nucleosome positioning sequences. Proc Natl Acad Sci U S A..

[35] Drew HR, Travers AA (1985). DNA bending and its relation to nucleosome positioning. J Mol Biol..

[36] Satchwell SC, Drew HR, Travers AA (1986). Sequence periodicities in chicken nucleosome core DNA. J Mol Biol..

[37] Lowman H, Bina M (1990). Correlation between dinucleotide periodicities and nucleosome positioning on mouse satellite DNA. Biopolymers..

[38] Segal E, Fondufe-Mittendorf Y, Chen L, Thastrom A, Field Y, Moore IK, Wang JP, Widom J (2006). A genomic code for nucleosome positioning. Nature..

[39] Bernstein BE, Liu CL, Humphrey EL, Perlstein EO, Schreiber SL (2004). Global nucleosome occupancy in yeast. Genome Biol..

[40] Lee C-K, Shibata Y, Rao B, Strahl BD, Lieb JD (2004). Evidence for nucleosome depletion at active regulatory regions genome-wide. Nat Genet..

[41] Lee W, Tillo D, Bray N, Morse RH, Davis RW, Hughes TR, Nislow C (2007). A high-resolution atlas of nucleosome occupancy in yeast. Nat Genet..

[42] Yuan G-C, Liu Y-J, Dion MF, Slack MD, Wu LF, Altschuler SJ, Rando OJ (2005). Genome-scale identification of nucleosome positions in S. cerevisiae. Science (New York).

[43] Jiang C, Pugh BF (2009). A compiled and systematic reference map of nucleosome positions across the Saccharomyces cerevisiae genome. Genome Biol..

[44] Kornberg RD, Stryer L (1988). Statistical distributions of nucleosomes: nonrandom locations by a stochastic mechanism. Nucleic Acids Res..

[45] Mavrich TN, Ioshikhes IP, Venters BJ, Jiang C, Tomsho LP, Qi J, Schuster SC, Albert I, Pugh BF (2008). A barrier nucleosome model for statistical positioning of nucleosomes throughout the yeast genome. Genome Res..

[46] Chereji RV, Tolkunov D, Locke G, Morozov AV (2011). Statistical mechanics of nucleosome ordering by chromatin-structure-induced two-body interactions. Phys Rev E Stat Nonlin Soft Matter Phys..

[47] Struhl K (1985). Naturally occurring poly(dA-dT) sequences are upstream promoter elements for constitutive transcription in yeast. Proc Natl Acad Sci U S A..

[48] Segal E, Widom J (2009). Poly(dA:dT) tracts: major determinants of nucleosome organization. Curr Opin Struct Biol..

[49] Schwabish MA, Struhl K (2004). Evidence for eviction and rapid deposition of histones upon transcriptional elongation by RNA polymerase II. Mol Cell Biol..

[50] Studitsky VM, Walter W, Kireeva M, Kashlev M, Felsenfeld G (2004). Chromatin remodeling by RNA polymerases. Trends Biochem Sci..

[51] Cole HA, Ocampo J, Iben JR, Chereji RV, Clark DJ (2014). Heavy transcription of yeast genes correlates with differential loss of histone H2B relative to H4 and queued RNA polymerases. Nucleic Acids Res..

[52] Fennessy RT, Owen-Hughes T (2016). Establishment of a promoter-based chromatin architecture on recently replicated DNA can accommodate variable inter-nucleosome spacing. Nucleic Acids Res..

[53] Ramachandran S, Henikoff S (2016). Transcriptional regulators compete with nucleosomes post-replication. Cell..

[54] Vasseur P, Tonazzini S, Ziane R, Camasses A, Rando OJ, Radman-Livaja M (2016). Dynamics of Nucleosome Positioning Maturation following Genomic Replication. Cell Rep..

[55] Yadav T, Whitehouse I (2016). Replication-coupled nucleosome assembly and positioning by ATP-dependent chromatin-remodeling enzymes. Cell Rep..

[56] Gkikopoulos T, Schofield P, Singh V, Pinskaya M, Mellor J, Smolle M, Workman JL, Barton GJ, Owen-Hughes T (2011). A role for Snf2-related nucleosome-spacing enzymes in genome-wide nucleosome organization. Science (New York).

[57] Zhang Z, Wippo CJ, Wal M, Ward E, Korber P, Pugh BF (2011). A packing mechanism for nucleosome organization reconstituted across a eukaryotic genome. Science (New York).

[58] Ganguli D, Chereji RV, Iben JR, Cole HA, Clark DJ (2014). RSC-dependent constructive and destructive interference between opposing arrays of phased nucleosomes in yeast. Genome Res..

[59] Musladin S, Krietenstein N, Korber P, Barbaric S (2014). The RSC chromatin remodeling complex has a crucial role in the complete remodeler set for yeast PHO5 promoter opening. Nucleic Acids Res..

[60] Ocampo J, Chereji RV, Eriksson PR, Clark DJ (2016). The ISW1 and CHD1 ATP-dependent chromatin remodelers compete to set nucleosome spacing in vivo. Nucleic Acids Res..

[61] Woo S, Zhang X, Sauteraud R, Robert F, Gottardo R (2013). PING 2.0: an R/Bioconductor package for nucleosome positioning using next-generation sequencing data. Bioinformatics.

[62] Zentner GE, Henikoff S (2013). Mot1 redistributes TBP from TATA-containing to TATA-less promoters. Mol Cell Biol..

[63] Zentner GE, Kasinathan S, Xin B, Rohs R, Henikoff S (2015). ChEC-seq kinetics discriminates transcription factor binding sites by DNA sequence and shape in vivo. Nat Commun..

[64] Parnell TJ, Schlichter A, Wilson BG, Cairns BR (2015). The chromatin remodelers RSC and ISW1 display functional and chromatin-based promoter antagonism. Elife..

[65] Hesselberth JR, Chen X, Zhang Z, Sabo PJ, Sandstrom R, Reynolds AP, Thurman RE, Neph S, Kuehn MS, Noble WS (2009). Global mapping of protein-DNA interactions in vivo by digital genomic footprinting. Nat Methods..

[66] Schep AN, Buenrostro JD, Denny SK, Schwartz K, Sherlock G, Greenleaf WJ (2015). Structured nucleosome fingerprints enable high-resolution mapping of chromatin architecture within regulatory regions. Genome Res..

[67] McGhee JD, Felsenfeld G (1983). Another potential artifact in the study of nucleosome phasing by chromatin digestion with micrococcal nuclease. Cell..

[68] Chereji RV, Ocampo J, Clark DJ (2017). MNase-Sensitive Complexes in Yeast: Nucleosomes and Non-histone Barriers. Mol Cell..

[69] Chereji RV, Kan T-W, Grudniewska MK, Romashchenko AV, Berezikov E, Zhimulev IF, Guryev V, Morozov AV, Moshkin YM (2016). Genome-wide profiling of nucleosome sensitivity and chromatin accessibility in Drosophila melanogaster. Nucleic Acids Res..

[70] Chereji RV, Morozov AV (2014). Ubiquitous nucleosome crowding in the yeast genome. Proc Natl Acad Sci U S A..

[71] Weiner A, Hughes A, Yassour M, Rando OJ, Friedman N (2010). High-resolution nucleosome mapping reveals transcription-dependent promoter packaging. Genome Res..

[72] Chereji RV, Morozov AV (2015). Functional roles of nucleosome stability and dynamics. Brief Funct Genomics..

[73] Churchman LS, Weissman JS (2011). Nascent transcript sequencing visualizes transcription at nucleotide resolution. Nature..

[74] Fan Y, Nikitina T, Zhao J, Fleury TJ, Bhattacharyya R, Bouhassira EE, Stein A, Woodcock CL, Skoultchi AI (2005). Histone H1 depletion in mammals alters global chromatin structure but causes specific changes in gene regulation. Cell..

[75] Oberg C, Izzo A, Schneider R, Wrange O, Belikov S (2012). Linker histone subtypes differ in their effect on nucleosomal spacing in vivo. J Mol Biol..

[76] Guillemette B, Bataille AR, Gévry N, Adam M, Blanchette M, Robert F, Gaudreau L (2005). Variant histone H2A.Z is globally localized to the promoters of inactive yeast genes and regulates nucleosome positioning. PLoS Biol.

[77] Zhang H, Roberts DN, Cairns BR (2005). Genome-wide dynamics of Htz1, a histone H2A variant that poises repressed/basal promoters for activation through histone loss. Cell..

[78] Elfving N, Chereji RV, Bharatula V, Björklund S, Morozov AV, Broach JR (2014). A dynamic interplay of nucleosome and Msn2 binding regulates kinetics of gene activation and repression following stress. Nucleic Acids Res..

[79] Qiu H, Chereji RV, Hu C, Cole HA, Rawal Y, Clark DJ, Hinnebusch AG (2016). Genome-wide cooperation by HAT Gcn5, remodeler SWI/SNF, and chaperone Ydj1 in promoter nucleosome eviction and transcriptional activation. Genome Res..

[80] Rhee HS, Bataille AR, Zhang L, Pugh BF (2014). Subnucleosomal structures and nucleosome asymmetry across a genome. Cell..

[81] Maltby VE, Martin BJE, Brind'Amour J, Chruscicki AT, McBurney KL, Schulze JM, Johnson IJ, Hills M, Hentrich T, Kobor MS (2012). Histone H3K4 demethylation is negatively regulated by histone H3 acetylation in Saccharomyces cerevisiae. Proc Natl Acad Sci U S A..

[82] Tonks L (1936). The complete equation of state of one, two and three-dimensional gases of hard elastic spheres. Phys Rev..

[83] Shivaswamy S, Bhinge A, Zhao Y, Jones S, Hirst M, Iyer VR (2008). Dynamic remodeling of individual nucleosomes across a eukaryotic genome in response to transcriptional perturbation. PLoS Biol..

[84] Noll M, Kornberg RD (1977). Action of micrococcal nuclease on chromatin and the location of histone H1. J Mol Biol..

[85] Van Holde KE (1989). Chromatin.

[86] Wang JP, Fondufe-Mittendorf Y, Xi L, Tsai GF, Segal E, Widom J (2008). Preferentially quantized linker DNA lengths in Saccharomyces cerevisiae. PLoS Comput Biol..

[87] Strauss F, Prunell A (1983). Organization of internucleosomal DNA in rat liver chromatin. EMBO J..

[88] Strauss F, Prunell A (1982). Nucleosome spacing in rat liver chromatin. A study with exonuclease III. Nucleic Acids Res.

[89] Prunell A, Kornberg RD (1982). Variable center to center distance of nucleosomes in chromatin. J Mol Biol..

[90] Morris NR (1976). Nucleosome structure in Aspergillus nidulans. Cell..

[91] Simpson RT, Bergman LW (1980). Structure of sea urchin sperm chromatin core particle. J Biol Chem..

[92] Perisic O, Collepardo-Guevara R, Schlick T (2010). Modeling studies of chromatin fiber structure as a function of DNA linker length. J Mol Biol..

[93] Norouzi D, Katebi A, Cui F, Zhurkin VB (2015). Topological diversity of chromatin fibers: Interplay between nucleosome repeat length, DNA linking number and the level of transcription. AIMS Biophysics..

[94] Locke G, Tolkunov D, Moqtaderi Z, Struhl K, Morozov AV (2010). High-throughput sequencing reveals a simple model of nucleosome energetics. Proc Natl Acad Sci U S A..

[95] Mobius W, Gerland U (2010). Quantitative test of the barrier nucleosome model for statistical positioning of nucleosomes up- and downstream of transcription start sites. PLoS Comput Biol..

[96] Krietenstein N, Wal M, Watanabe S, Park B, Peterson CL, Pugh BF, Korber P (2016). Genomic nucleosome organization reconstituted with pure proteins. Cell..

[97] Nagarajavel V, Iben JR, Howard BH, Maraia RJ, Clark DJ (2013). Global 'bootprinting' reveals the elastic architecture of the yeast TFIIIB-TFIIIC transcription complex in vivo. Nucleic Acids Res..

[98] Belsky JA, MacAlpine HK, Lubelsky Y, Hartemink AJ, MacAlpine DM (2015). Genome-wide chromatin footprinting reveals changes in replication origin architecture induced by pre-RC assembly. Genes Dev..

[99] Dai J, Hyland EM, Yuan DS, Huang H, Bader JS, Boeke JD (2008). Probing nucleosome function: a highly versatile library of synthetic histone H3 and H4 mutants. Cell..

[100] Langmead B, Salzberg SL (2012). Fast gapped-read alignment with Bowtie 2. Nat Methods..

[101] Robinson JT, Thorvaldsdottir H, Winckler W, Guttman M, Lander ES, Getz G, Mesirov JP (2011). Integrative genomics viewer. Nat Biotechnol..

[102] Park D, Morris AR, Battenhouse A, Iyer VR (2014). Simultaneous mapping of transcript ends at single-nucleotide resolution and identification of widespread promoter-associated non-coding RNA governed by TATA elements. Nucleic Acids Res..

[103] Pathria RK, Beale PD (2011). Statistical Mechanics.

[104] Chereji RV, Ramachandran S, Bryson TD, Henikoff S. Precise genome-wide mapping of single nucleosomes and linkers in vivo. Gene Expression Omnibus. 2018, GSE97290: https://www.ncbi.nlm.nih.gov/geo/query/acc.cgi?acc=GSE97290. 10.1186/s13059-018-1398-0 PMC5807854 29426353

[105] Chereji RV (2013). Statistical mechanics of nucleosomes.

