# Supplementary material for: Precise genome-wide mapping of single nucleosomes and linkers in vivo
Source: Genome Biol. 2018 Feb 9;19:19. doi: 10.1186/s13059-018-1398-0 (PMC5807854; doi:10.1186/s13059-018-1398-0)
Supplement: Supplementary file 1 — Chemical cleavages produced by H3Q85C. Figure S2. H3Q85C cleavage mapping distinguishes between real NDRs and MNase artifacts (MNase-sensitive nucleosomes). Figure S3. Determination of the gap size using cross-correlations between the distributions of the DNA fragment ends. Figure S4. Correlations among nucleosome spacing, NDR width, transcription levels, and ChIP-seq data. Figure S5. Sketch of the statistical mechanics formalism for predicting nucleosome occupancy. (PDF 2035 kb) [file 13059_2018_1398_MOESM1_ESM.pdf]

Supplementary Information

Precise genome-wide mapping of single nucleosomes and linkers *in vivo*

Răzvan V. Chereji[1,3], Srinivas Ramachandran[2,3], Terri D. Bryson[2], and Steven Henikoff[2,*]

[1]Program in Genomics of Differentiation, *Eunice Kennedy Shriver* National Institute for Child Health and Human Development, National Institutes of Health, Bethesda, Maryland 20892, USA
[2]Howard Hughes Medical Institute and Basic Sciences Division, Fred Hutchinson Cancer Research Center, Seattle, WA 98109, USA
[3]These authors contributed equally to this work.
[*]Corresponding author. E-mail: steveh@fhcrc.org

Contents

Supplementary Figures **3**
Supplementary Figure S1 . . . 3
Supplementary Figure S2 . . . 4
Supplementary Figure S3 . . . 5
Supplementary Figure S4 . . . 6
Supplementary Figure S5 . . . 7

Supplementary Tables **8**

Supplementary References **9**

Supplementary Figures

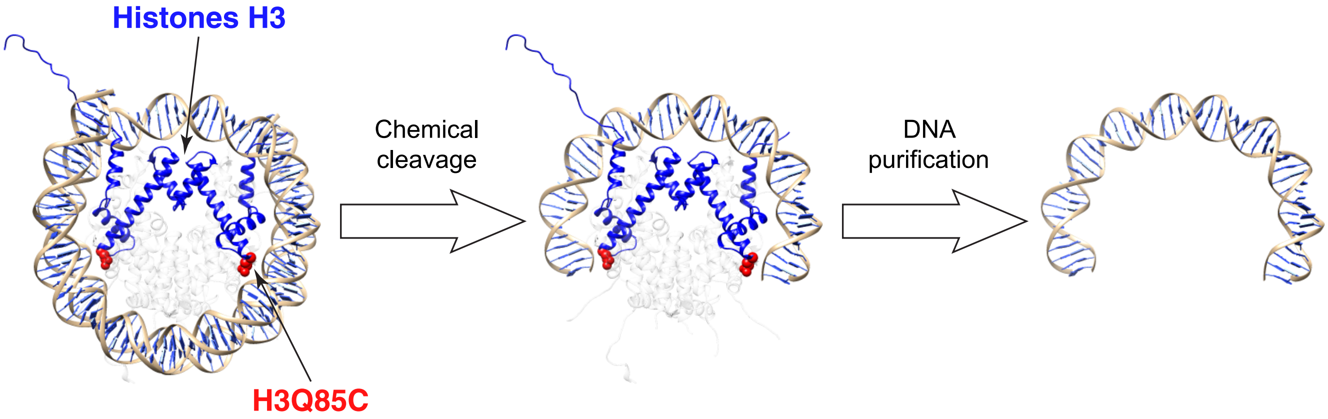

Supplementary Figure S1. Chemical cleavages produced by H3Q85C. Crystal structure of the nucleosome (PDB: 1AOI) (Luger et al., 1997), with the histones H3 colored in blue, and the mutated amino-acid, glutamine 85, shown in red. The introduced cysteines are located next to nucleosomal DNA, and produce cleavages of the sugar-phosphate backbone, which will result in DNA fragments of $\sim$51 bp, symmetrical relative to the nucleosome dyad axis. The midpoint of each $\sim$51 bp fragment precisely identifies the position of the nucleosome center, without relying on any statistical inference procedures. The background generated by the cleavages produced due to free phenanthroline is drastically reduced compared to the background produced by H4S47C, as we require two concurrent cleavages, separated by $\sim$51 bp, in order to infer a valid nucleosome position.

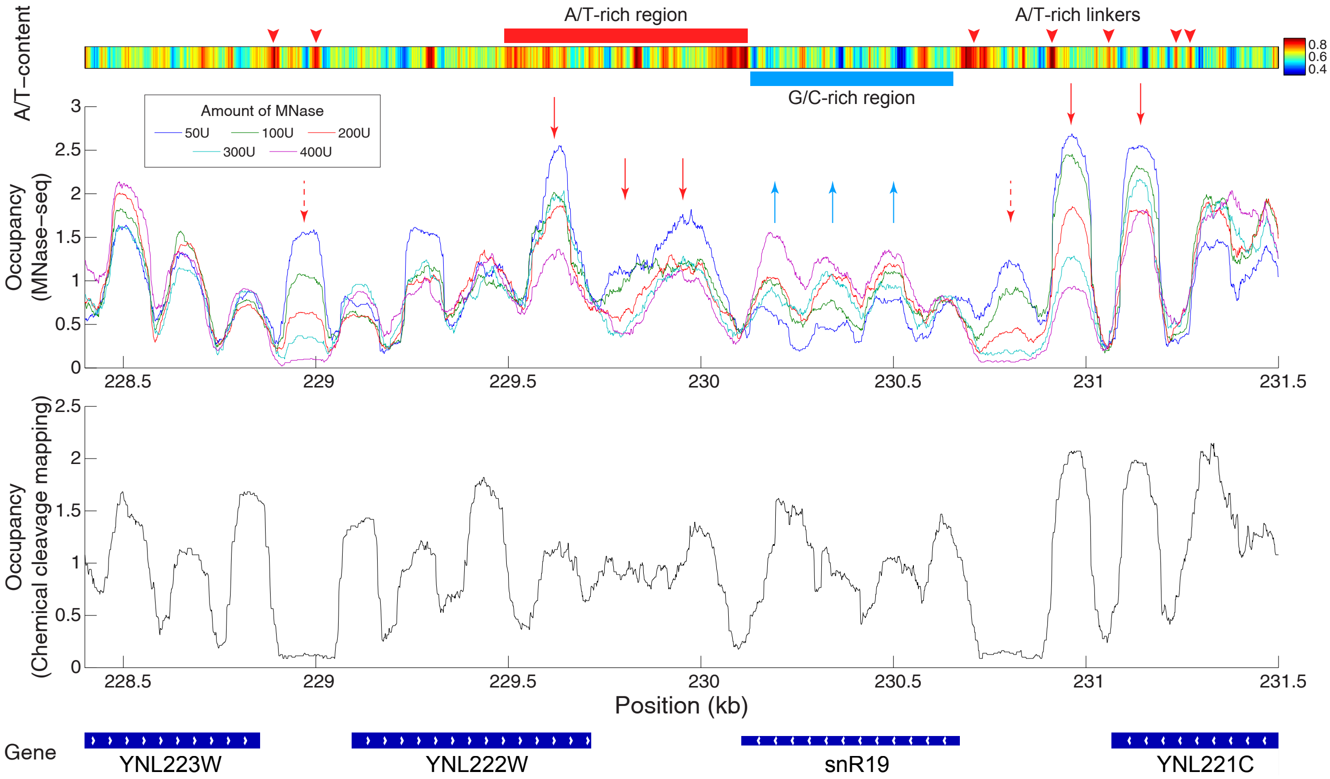

Supplementary Figure S2. H3Q85C cleavage mapping distinguishes between real NDRs and MNase artifacts (MNase-sensitive nucleosomes). Nucleosome occupancy obtained by MNase-seq is affected by MNase biases. During an MNase titration, the measured occupancy can increase (blue upward arrows) or decrease (red downward arrows). Usually, nucleosomes with A/T-rich linkers (red arrowheads), or located in a broad A/T-rich region (red rectangle) are digested faster, and are under-represented in the extensively-digested samples. Nucleosomes from A/T-poor regions (blue rectangle) are under-represented in the mildly digested samples, as these are harder to release from chromatin in the initial stages of the MNase digestion. The chemical cleavage method can distinguish between MNase-sensitive nucleosomes (*e.g.* the ones between YNL222W and snR19 and the two nucleosomes near the TTS of YNL221C, marked by solid red downward arrows) and the real NDRs (marked with dashed red downward arrows), eliminating the bias introduced by MNase. The occupancy profiles were generated as in Figure 1.

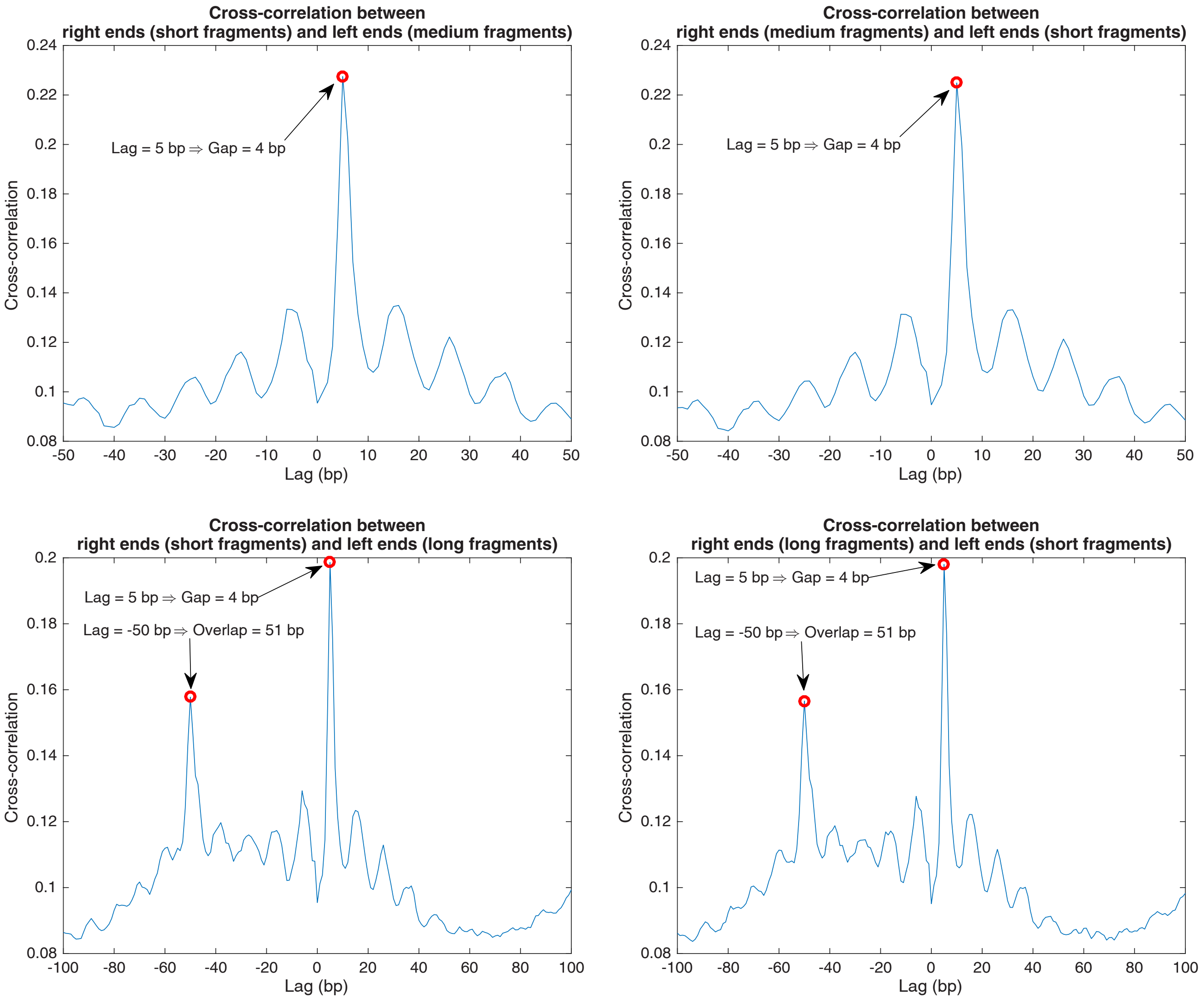

Supplementary Figure S3. Determination of the gap size using cross-correlations between the distributions of the DNA fragment ends. Cross-correlation between the distributions of the fragment ends that originate from the same cleavage site (see Fig. 5) allows us to estimate the gap size of 4 bp. Cross-correlation `r` was computed in MATLAB using the `xcorr` function, a maximum lag of 100 bp, and the normalization option `'coeff'`: `[r, lags] = xcorr(x, y, 100, 'coeff')`, where `x` and `y` represent the distributions indicated above each panel. Cross-correlation `r(d)` measures the similarity between `x` and shifted (lagged) copies of `y` as a function of the lag `d`.

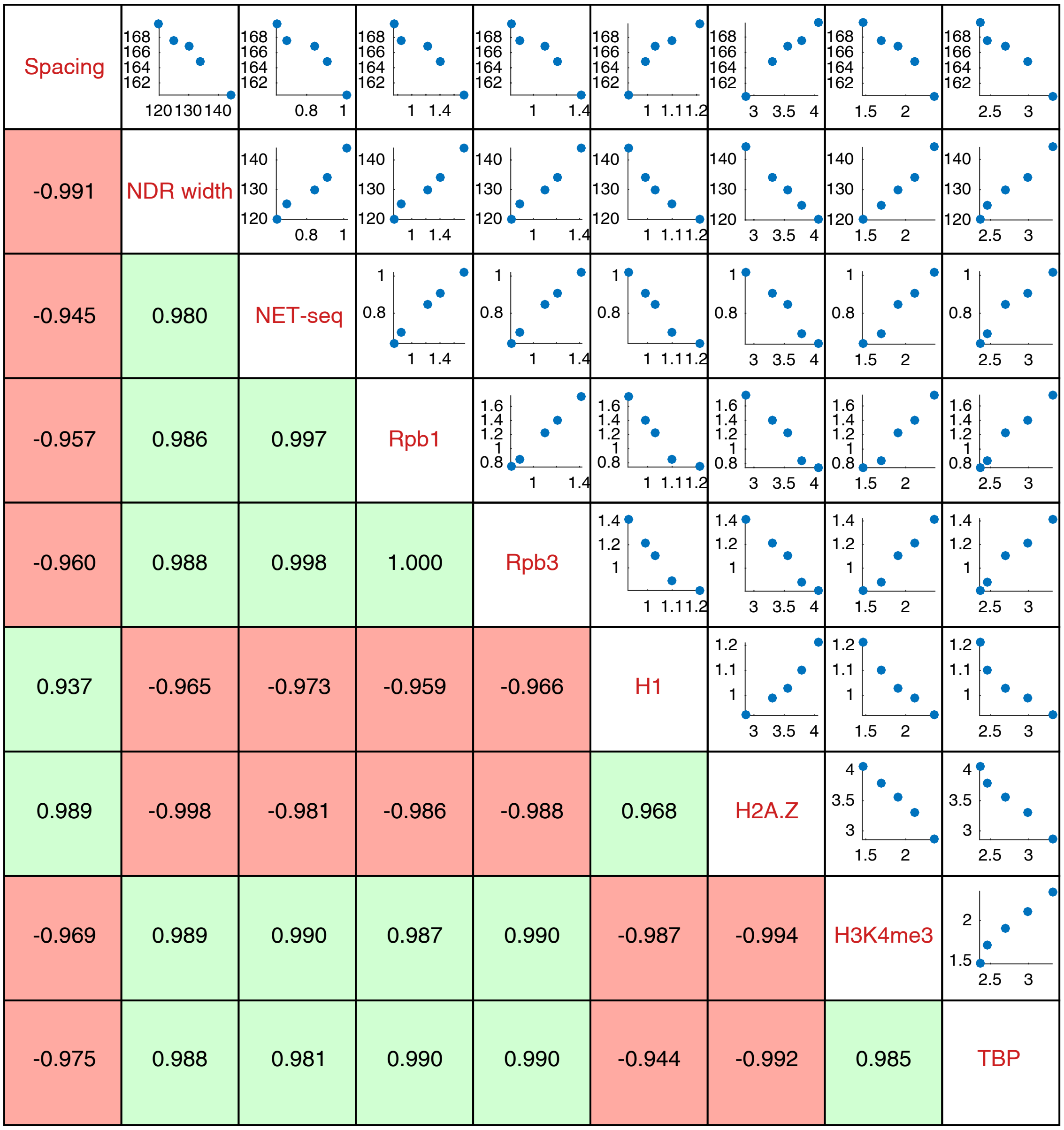

Supplementary Figure S4. Correlations among nucleosome spacing, NDR width, transcription levels, and ChIP-seq data. Yeast genes were split into five quintiles according to the corresponding nucleosome spacing, estimated from the average length of the long DNA fragments (135–175 bp) resulted after the chemical cleavage. Above the main diagonal: Scatter plots with the quintile averages of the quantities shown in Figure 7D: average nucleosome spacing and average NDR width, estimated from Figure 7B; average NET-seq signal on the gene bodies (data from Churchman and Weissman (2011)); average Rpb1 density on the gene bodies (data from Elfving et al. (2014)); average Rpb3 density on the gene bodies (data from Qiu et al. (2016)); average H1 cross-link density in the window $[D-73, D+750]$ bp, where D is the dyad position for +1 nucleosome (data from Rhee et al. (2014)); average H2A.Z occupancy for the +1 nucleosomes (data from Woo et al. (2013)); average H3K4me3 occupancy computed in the same range as for H1 density (data from Maltby et al. (2012)); average TBP occupancy in the window $[D-500, D-73]$ bp, where D is the dyad position for +1 nucleosome (data from Zentner and Henikoff (2013)). Below the main diagonal: Pearson correlation coefficients among the values represented in the corresponding scatter plots. Green squares indicate a good correlation, while red squares indicate a good anti-correlation.

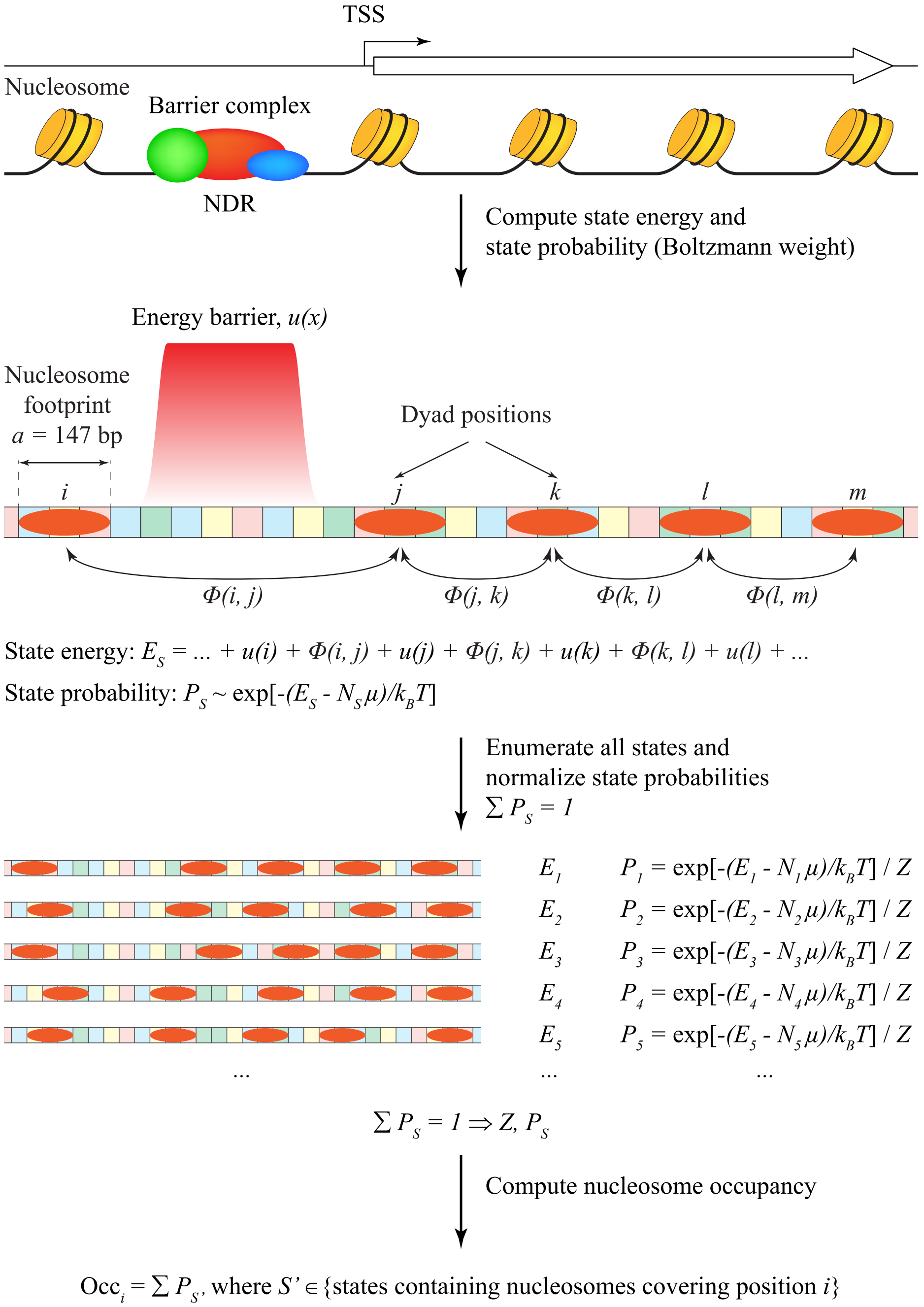

Supplementary Figure S5. Sketch of the statistical mechanics formalism for predicting nucleosome occupancy. Each chromosome is modeled as a one-dimensional lattice, where nucleosomes, modeled as hard rods, can occupy any non-overlapping positions. The effect of non-histone barrier complex from promoters is modeled as an external energy barrier. For each state, S, the energy of the state, E_S, will dictate its probability, P_S, according to a Boltzmann weight. The normalization factor of the state probabilities or the partition function, Z, can be computed by summing the Boltzmann weights of all valid states, in a recursive way Chereji et al. (2016). After the state probabilities are normalized, the probability of having a nucleosome covering the position i (nucleosome occupancy) is computed by simply summing the probabilities for all states where a nucleosome is covering position i.

Supplementary Tables

Supplementary Table S1. Coordinates of the dominant positions of +1 and −1 nucleosomes identified in this study.

Supplementary References

Chereji RV, Kan TW, Grudniewska MK, Romashchenko AV, Berezikov E, Zhimulev IF, Guryev V, Morozov AV, and Moshkin YM. 2016. Genome-wide profiling of nucleosome sensitivity and chromatin accessibility in Drosophila melanogaster. *Nucleic Acids Res.* **44**: 1036–1051.

Churchman LS and Weissman JS. 2011. Nascent transcript sequencing visualizes transcription at nucleotide resolution. *Nature* **469**: 368–373.

Elfving N, Chereji RV, Bharatula V, Björklund S, Morozov AV, and Broach JR. 2014. A dynamic interplay of nucleosome and Msn2 binding regulates kinetics of gene activation and repression following stress. *Nucleic Acids Res.* **42**: 5468–5482.

Luger K, Mäder AW, Richmond RK, Sargent DF, and Richmond TJ. 1997. Crystal structure of the nucleosome core particle at 2.8 å resolution. *Nature* **389**: 251–260.

Maltby VE, Martin BJ, BrindAmour J, Chruscicki AT, McBurney KL, Schulze JM, Johnson IJ, Hills M, Hentrich T, Kobor MS, et al.. 2012. Histone H3K4 demethylation is negatively regulated by histone H3 acetylation in Saccharomyces cerevisiae. *Proc. Natl. Acad. Sci. U.S.A.* **109**: 18505–18510.

Qiu H, Chereji RV, Hu C, Cole HA, Rawal Y, Clark DJ, and Hinnebusch AG. 2016. Genome-wide cooperation by HAT Gcn5, remodeler SWI/SNF, and chaperone Ydj1 in promoter nucleosome eviction and transcriptional activation. *Genome Res.* **26**: 211–225.

Rhee HS, Bataille AR, Zhang L, and Pugh BF. 2014. Subnucleosomal structures and nucleosome asymmetry across a genome. *Cell* **159**: 1377–1388.

Woo S, Zhang X, Sauteraud R, Robert F, and Gottardo R. 2013. PING 2.0: an R/Bioconductor package for nucleosome positioning using next-generation sequencing data. *Bioinformatics* **29**: 2049–2050.

Zentner GE and Henikoff S. 2013. Mot1 redistributes TBP from TATA-containing to TATA-less promoters. *Mol. Cell. Biol.* **33**: 4996–5004.
